# Sampling and sensitivity analyses tools (SaSAT) for computational modelling

**DOI:** 10.1186/1742-4682-5-4

**Published:** 2008-02-27

**Authors:** Alexander Hoare, David G Regan, David P Wilson

**Affiliations:** 1 National Centre in HIV Epidemiology and Clinical Research, The University of New South Wales, Sydney, New South Wales, 2010, Australia

## Abstract

SaSAT (Sampling and Sensitivity Analysis Tools) is a user-friendly software package for applying uncertainty and sensitivity analyses to mathematical and computational models of arbitrary complexity and context. The toolbox is built in Matlab^®^, a numerical mathematical software package, and utilises algorithms contained in the Matlab^® ^Statistics Toolbox. However, Matlab^® ^is not required to use SaSAT as the software package is provided as an executable file with all the necessary supplementary files. The SaSAT package is also designed to work seamlessly with Microsoft Excel but no functionality is forfeited if that software is not available. A comprehensive suite of tools is provided to enable the following tasks to be easily performed: efficient and equitable sampling of parameter space by various methodologies; calculation of correlation coefficients; regression analysis; factor prioritisation; and graphical output of results, including response surfaces, tornado plots, and scatterplots. Use of SaSAT is exemplified by application to a simple epidemic model. To our knowledge, a number of the methods available in SaSAT for performing sensitivity analyses have not previously been used in epidemiological modelling and their usefulness in this context is demonstrated.

## Introduction

Mathematical and computational models today play a key role in almost every branch of science. The rapid advances in computer technology have led to increasingly more complex models as performance more like the real systems being investigated is sought. As a result, uncertainty and sensitivity analyses for quantifying the range of variability in model responses and for identifying the key factors giving rise to model outcomes have become essential for determining model robustness and reliability and for ensuring transparency [1]. Furthermore, as it is not uncommon for models to have dozens or even hundreds of independent predictors, these analyses usually constitute the first and primary approach for establishing mechanistic insights to the observed responses.

The challenge in conducting uncertainty analysis for models with moderate to large numbers of parameters is to explore the multi-dimensional parameter space in an equitable and computationally efficient way. Latin hypercube sampling (LHS), a type of stratified Monte Carlo sampling [2,3] that is an extension of Latin Square sampling [4,5] first proposed by McKay at al. [6] and further developed and introduced by Iman et al. [1-3], is a sophisticated and efficient method for achieving equitable sampling of all predictors simultaneously. Uncertainty analyses in this context use parameter samples generated by LHS as inputs in an independent external model; each sample may produce a different model response/outcome. Sensitivity analysis may then be conducted to rank the predictors (input parameters) in terms of their contribution to the uncertainty in each of the responses (model outcomes). This can be achieved in several ways involving primarily the calculation of correlation coefficients and regression analysis [1,7], and variance-based methods [8].

In response to our need to conduct these analyses for numerous and diverse modelling exercises, we were motivated to develop a suite of tools, assembled behind a user-friendly interface, that would facilitate this process. We have named this toolbox SaSAT for "Sampling and Sensitivity Analysis Tools". The toolbox was developed in the widely used mathematical software package Matlab^® ^(The Mathworks, Inc., MA, USA) and utilises the industrial strength algorithms built into this package and the Matlab^® ^Statistics Toolbox. It enables uncertainty analysis to be applied to models of arbitrary complexity, using the LHS method for sampling the input parameter space. SaSAT is independent of the model being applied; SaSAT generates input parameter samples for an external model and then uses these samples in conjunction with outputs (responses) generated from the external model to perform sensitivity analyses. A variety of methods are available for conducting sensitivity analyses including the calculation of correlation coefficients, standardised and non-standardised linear regression, logistic regression, Kolmogorov-Smirnov test, and factor prioritization by reduction of variance. The option to import data from, and export data to, Microsoft Excel or Matlab^® ^is provided but not requisite. The results of analyses can be output in a variety of graphical and text-based formats.

While the utility of the toolbox is not confined to any particular discipline or modelling paradigm, the last two or three decades have seen remarkable growth in the use and importance of mathematical modelling in the epidemiological context (the primary context for modelling by the authors). However, many of the methods for uncertainty and sensitivity analysis that have been used extensively in other disciplines have not been widely used in epidemiological modelling. This paper provides a description of the SaSAT toolbox and the methods it employs, and exemplifies its use by application to a simple epidemic model with intervention. But SaSAT can be used in conjunction with theoretical or computational models applied to any discipline. Online supplementary material to this paper provides the freely downloadable full version of the SaSAT software for use by other practitioners [see Additional file 1].

## Description of methods

In this section we provide a very brief overview and description of the sampling and sensitivity analysis methods used in SaSAT. A user manual for the software is provided as supplementary material. Note that we use the terms parameter, predictor, explanatory variable, factor interchangeably, as well as outcome, output variable, and response.

### Sampling methods and uncertainty analysis

Uncertainty analyses explore parameter ranges rather than simply focusing on specific parameter values. They are used to determine the degree of uncertainty in model outcomes that is due to uncertainty in the input parameters. Each input parameter for a model can be defined to have an appropriate probability density function associated with it. Then, the computational model can be simulated by sampling a single value from each parameter's distribution. Many samples should be taken and many simulations should be run, producing variable output values. The variation in the output can then be explored as it relates to the variation in the input. There are various approaches that could be taken to sample from the parameter distributions. Ideally one should vary all (*M*) model parameters simultaneously in the *M*-dimensional parameter space in an efficient manner. SaSAT provides random sampling, full factorial sampling, and Latin Hypercube Sampling.

#### Random sampling

**Figure 1:**
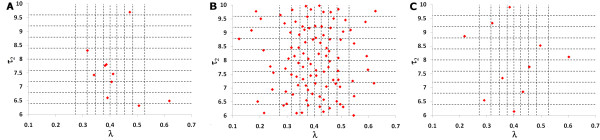
Examples of the three different sampling schemes: **(a) **random sampling, **(b) **full factorial sampling, and **(c) **Latin Hypercube Sampling, for a simple case of 10 samples (samples for *τ*_2 _~ *U *(6,10) and *λ *~ *N *(0.4, 0.1) are shown). In random sampling, there are regions of the parameter space that are not sampled and other regions that are heavily sampled; in full factorial sampling, a random value is chosen in each interval for each parameter and every possible combination of parameter values is chosen; in Latin Hypercube Sampling, a value is chosen once and only once from every interval of every parameter (it is efficient and adequately samples the entire parameter space).

The first obvious sampling approach is random sampling whereby each parameter's distribution is used to draw *N *values randomly. This is generally vastly superior to univariate approaches to uncertainty and sensitivity analyses, but it is not the most efficient way to sample the parameter space. In Figure 1a we present one instance of random sampling of two parameters.

#### Full factorial sampling

The full factorial sampling scheme uses a value from every sampling interval for each possible combination of parameters (see Figure 1b for an illustrative example). This approach has the advantage of exploring the entire parameter space but is extremely computationally inefficient and time-consuming and thus not feasible for all models. If there are *M *parameters and each one has *N *values (or its distribution is divided into *N *equiprobable intervals), then the total number of parameter sets and model simulations is *N*^*M *^(for example, 20 parameters and 100 samples per distribution would result in 10^40 ^unique combinations, which is essentially unfeasible for most practical models). However, on occasion full factorial sampling can be feasible and useful, such as when there are a small number of parameters and few samples required.

#### Latin hypercube sampling

**Figure 2:**
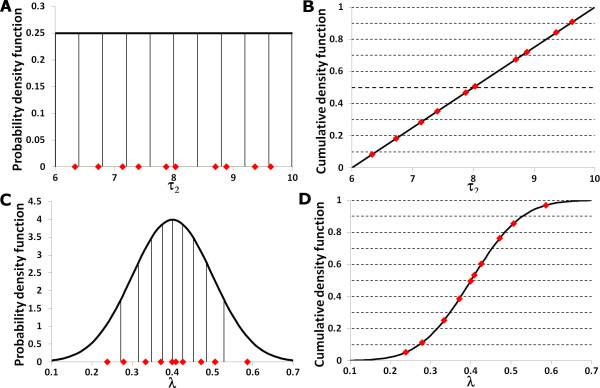
Examples of the probability density functions (**(a) **and **(c)**) and cumulative density functions (**(b) **and **(d)**) associated with parameters used in Figure 1; the black vertical lines divide the probability density functions into areas of equal probability. The red diamonds depict the location of the samples taken. Since these samples are generated using Latin Hypercube sampling there is one sample for each area of equal probability. The example distributions are: **(a) **A uniform distribution of the parameter *τ*_2_, **(b) **the cumulative density function of *τ*_2_, **(c) **a normal distribution function for the parameter *λ*, and **(d) **cumulative density function of *λ*.

More efficient and refined statistical techniques have been applied to sampling. Currently, the standard sampling technique employed is Latin Hypercube Sampling and this was introduced to the field of disease modelling (the field of our research) by Blower [9]. For each parameter a probability density function is defined and stratified into *N *equiprobable serial intervals. A single value is then selected randomly from every interval and this is done for every parameter. In this way, an input value from each sampling interval is used only once in the analysis but the entire parameter space is equitably sampled in an efficient manner [1,9-11]. Distributions of the outcome variables can then be derived directly by running the model *N *times with each of the sampled parameter sets. The algorithm for the Latin Hypercube Sampling methodology is described clearly in [9]. Figure 1c and Figure 2 illustrate how the probability density functions are divided into equiprobable intervals and provide an example of the sampling.

#### Sensitivity analyses for continuous variables

Sensitivity analysis is used to determine how the uncertainty in the output from computational models can be apportioned to sources of variability in the model inputs [9,12]. A good sensitivity analysis will extend an uncertainty analysis by identifying which parameters are important (due to the variability in their uncertainty) in contributing to the variability in the outcome variable [1]. A description of the sensitivity analysis methods available in SaSAT is now provided.

#### Correlation coefficients

The association, or relationship, between two different kinds of variables or measurements is often of considerable interest. The standard measure of ascertaining such associations is the correlation coefficient; it is given as a value between -1 and +1 which indicates the degree to which two variables (e.g., an input parameter and output variable) are linearly related. If the relationship is perfectly linear (such that all data points lie perfectly on a straight line), the correlation coefficient is +1 if there is a positive correlation and -1 if the line has a negative slope. A correlation coefficient of zero means that there is no linear relationship between the variables. SaSAT provides three types of correlation coefficients, namely: Pearson; Spearman; and Partial Rank. These correlation coefficients depend on the variability of variables. Therefore it should be noted that if a predictor is highly important but has only a single point estimate then it will not have correlation with outcome variability, but if it is given a wide uncertainty range then it may have a large correlation coefficient (if there is an association). Raw samples can be used in these analyses and do not need to be standardized.

Interpretation of the Pearson correlation coefficient assumes both variables follow a Normal distribution and that the relationship between the variables is a linear one. It is the simplest of correlation measures and is described in all basic statistics textbooks [13]. When the assumption of normality is not justified, and/or the relationship between the variables is non-linear, a non-parametric measure such as the Spearman Rank Correlation Coefficient is more appropriate. By assigning ranks to data (positioning each datum point on an ordinal scale in relation to all other data points), any outliers can also be incorporated without heavily biasing the calculated relationship. This measure assesses how well an arbitrary monotonic function describes the relationship between two variables, without making any assumptions about the frequency distribution of the variables. Such measures are powerful when only a single pair of variables is to be investigated. However, quite often measurements of different kinds will occur in batches. This is especially the case in the analysis of most computational models that have many input parameters and various outcome variables. Here, the relationship between each input parameter with each outcome variable is desired. Specifically, each relationship should be ascertained whilst also acknowledging that there are various other contributing factors (input parameters). Simple correlation analyses could be carried out by taking the pairing of each outcome variable and each input parameter in turn, but it would be unwieldy and would fail to reveal more complicated patterns of relationships that might exist between the outcome variables and several variables simultaneously. Therefore, an extension is required and the appropriate extension for handling groups of variables is partial correlation. For example, one may want to know how A was related to B when controlling for the effects of C, D, and E. Partial rank correlation coefficients (PRCCs) are the most general and appropriate method in this case. We recommend calculating PRCCs for most applications. The method of calculating PRCCs for the purpose of sensitivity analysis was first developed for risk analysis in various systems [2-5,14]. Blower pioneered its application to disease transmission models [9,15-22]. Because the outcome variables of dynamic models are time dependent, PRCCs should be calculated over the outcome time-course to determine whether they also change substantially with time. The interpretation of PRCCs assumes a monotonic relationship between the variables. Thus, it is also important to examine scatter-plots of each model parameter versus each predicted outcome variable to check for monotonicity and discontinuities [4,9,23]. PRCCs are useful for identifying the most important parameters but not for quantifying how much change occurs in the outcome variable by changing the value of the input parameter. However, because they have a sign (positive or negative) PRCCs can indicate the direction of change in the outcome variable if there is an increase or decrease in the input parameter. This can be further explored with regression and response surface analyses.

#### Regression

When the relationship between variables is not monotonic or when measurements are arbitrarily or irregularly distributed, regression analysis is more appropriate than simple correlation coefficients. A regression equation provides an expression of the relationship between two (or more) variables algebraically and indicates the extent to which a dependent variable can be predicted by knowing the values of other variables, or the extent of the association with other variables. In effect, the regression model is a surrogate for the true computational model. Accordingly, the coefficient of determination, *R*^2^, should be calculated with all regression models and the regression analysis should not be used if *R*^2 ^is low (arbitrarily, less than ~ 0.6). *R*^2 ^indicates the proportion of the variability in the data set that is explained by the fitted model and is calculated as the ratio of the sum of squares of the residuals to the total sum of squares. The adjusted *R*^2 ^statistic is a modification of *R*^2 ^that adjusts for the number of explanatory terms in the model. *R*^2 ^will tend to increase with the number of terms in the statistical model and therefore cannot be used as a meaningful comparator of models with different numbers of covariants (e.g., linear versus quadratic). The adjusted *R*^2^, however, increases only if the new term improves the model more than would be expected by chance and is therefore preferable for making such comparisons. Both *R*^2 ^and adjusted *R*^2 ^measures are provided in SaSAT.

Regression analysis seeks to relate a response, or output variable, to a number of predictors or input variables that affect it. Although higher-order polynomial expressions can be used, constructing linear regression equations with interaction terms or full quadratic responses is recommended. This is in order to include direct effects of each input variable and also variable cross interactions and nonlinearities; that is, the effect of each input variable is directly accounted for by linear terms as a first-order approximation but we also include the effects of second-order nonlinearities associated with each variable and possible interactions between variables. The generalized form of the full second-order regression model is:

$$Y=\beta_{0}+\sum_{i=1}^{m}\beta_{i}X_{i}+\sum_{i=1}^{m}\beta_{ii}X_{i}^{2}+\sum_{i=1}^{m-1}\sum_{j=i+1}^{m}\beta_{ij}X_{i}X_{j},$$

where *Y *is the dependent response variable, the *X*_*i*_'s are the predictor (input parameter) variables, and the *β*'s are regression coefficients.

One of the values of regression analysis is that results can be inspected visually. If there is only a single explanatory input variable for an outcome variable of interest, then the regression equation can be plotted graphically as a curve; if there are two explanatory variables then a three dimensional surface can be plotted. For greater than two explanatory variables the resulting regression equation is a hypersurface. Although hypersurfaces cannot be shown graphically, contour plots can be generated by taking level slices, fixing certain parameters. Further, complex relationships and interactions between outputs and input parameters are simplified in an easily interpreted manner [24,25]. Cross-products of input parameters reveal interaction effects of model input parameters, and squared or higher order terms allow curvature of the hypersurface. Obviously this can best be presented and understood when the dominant two predicting parameters are used so that the hypersurface is a visualised surface.

Although regression analysis can be useful to predict a response based on the values of the explanatory variables, the coefficients of the regression expression do not provide mechanistic insight nor do they indicate which parameters are most influential in affecting the outcome variable. This is due to differences in the magnitudes and variability of explanatory variables, and because the variables will usually be associated with different units. These are referred to as unstandardized variables and regression analysis applied to unstandardized variables yields unstandardized coefficients. The independent and dependent variables can be standardized by subtracting the mean and dividing by the standard deviation of the values of the unstandardized variables yielding standardized variables with mean of zero and variance of one. Regression analysis on standardized variables produces standardized coefficients [26], which represent the change in the response variable that results from a change of one standard deviation in the corresponding explanatory variable. While it must be noted that there is no reason why a change of one standard deviation in one variable should be comparable with one standard deviation in another variable, standardized coefficients enable the order of importance of the explanatory variables to be determined (in much the same way as PRCCs). Standardized coefficients should be interpreted carefully – indeed, unstandardized measures are often more informative. Standardized coefficients take values between -1 and +1; a standardized coefficient of +/-1 means that the predictor variable perfectly describes the response variable and a value of zero means that the predictor variable has no influence in predicting the response variable. Standardized regression coefficients should not, however, be considered to be equivalent to PRCCs. They both take values in the same range (-1 to +1), can be used to rank parameter importance, and have similar interpretations at the extremes but they are evaluated differently and measure different quantities. Consequently, PRCCs and standardized regression coefficients will differ in value and may differ slightly in ranking when analysing the same data. The magnitude of standardized regression coefficients will typically be lower than PRCCs and should not be used alone for determining variable importance when there are large numbers of explanatory variables. However, the regression equation can provide more meaningful sensitivity than correlation coefficients as it can be shown that an x% decrease in one parameter can be offset by a y% increase/decrease in another, simply by exploring the coefficients of the regression equation. It must be noted that this is true for the statistical model, which is a surrogate for the actual model. The degree to which such claims can be inferred to the true model is determined by the coefficient of determination, *R*_2_.

#### Factor prioritization by reduction of variance

Factor prioritization is a broad term denoting a group of statistical methodologies for ranking the importance of variables in contributing to particular outcomes. Variance-based measures for factor prioritization have yet to be used in many computational modelling fields,, although they are popular in some disciplines [27-34]. The objective of reduction of variance is to identify the factor which, if determined (that is, fixed to its true, albeit unknown, value), would lead to the greatest reduction in the variance of the output variable of interest. The second most important factor in reducing the outcome is then determined etc., until all independent input factors are ranked. The concept of importance is thus explicitly linked to a reduction of the variance of the outcome. Reduction of variance can be described conceptually by the following question: for a generic model,

*Y *= *f*(*X*_1_,...,*X*_*M*_),

how would the uncertainty in *Y *change if a particular independent variable *X*_*i *_could be fixed as a constant? This resultant variation is denoted by *V*_**X **~ *i*_(*Y*|*X*_*i *_= $x_{i}^{*}$). We expect that having fixed one source of variation (*X*_*i*_), the resulting variance *V*_**X**~*i*_(*Y*|*X*_*i *_= $x_{i}^{*}$) would be smaller than the total or unconditional variance *V*(*Y*). Hence, *V*_**X**~*i*_(*Y*|*X*_*i *_= $x_{i}^{*}$) can be used as a measure of the importance of *X*_*i *_; the smaller *V*_**X**~*i*_(*Y*|*X*_*i *_= $x_{i}^{*}$), the more *X*_*i *_is influential. However, this is based on sensitivity with respect to the position of a single point *X*_*i *_= $x_{i}^{*}$ for each input variable, and it is also possible to design a model for which *V*_**X**~*i*_(*Y*|*X*_*i *_= $x_{i}^{*}$) at particular $x_{i}^{*}$ values is greater than the unconditional variance, *V*(*Y*) [35]. In general, it is also not possible to obtain a precise factor prioritization, as this would imply knowing the true value of each factor. The reduction of variance methodology is therefore applied to rank parameters in terms of their direct contribution to uncertainty in the outcome. The factor of greatest importance is determined to be that, which when fixed, will on average result in the greatest reduction in variance in the outcome. "On average" specifies in this case that the variation of the outcome factor should be averaged over the defined distribution of the specific input factor, removing the dependence on $x_{i}^{*}$. This is written as $E_{X_{i}}\left(V_{X\sim i}\left(Y|X_{i}\right)\right)$ and will always be less than or equal to *V*(*Y*); in fact,

$$E_{X_{i}}\left(V_{X\sim i}\left(Y|X_{i}\right)\right)+V_{X_{i}}\left(E_{X\sim i}\left(Y|X_{i}\right)\right)=V(Y).$$

A small $E_{X_{i}}\left(V_{X\sim i}\left(Y|X_{i}\right)\right)$, or a large $V_{X_{i}}\left(E_{X\sim i}\left(Y|X_{i}\right)\right)$ implies that *X*_*i *_is an important factor. Then, a first order sensitivity index of *X*_*i *_on *Y *can be defined as

$$S_{i}=\frac{V_{X_{i}}\left(E_{X\sim i}\left(Y|X_{i}\right)\right)}{V(Y)}.$$

Conveniently, the sensitivity index takes values between 0 and 1. A high value of *S*_*i *_implies that *X*_*i *_is an important variable. Variance based measures, such as the sensitivity index just defined, are concise, and easy to understand and communicate. This is an appropriate measure of sensitivity to use to rank the input factors in order of importance even if the input factors are correlated [36]. Furthermore, this method is completely 'model-free'. The sensitivity index is also very easy to interpret; *S*_*i *_can be interpreted as being the proportion of the total variance attributable to variable *X*_*i*_. In practice, this measure is calculated by using the input variables and output variables and fitting a surrogate model, such as a regression equation; a regression model is used in our SaSAT application. Therefore, one must check that the coefficient of determination is sufficiently large for this method to be reliable (an *R*^2 ^value for the chosen regression model can be calculated in SaSAT).

#### Sensitivity analyses for binary outputs: logistic regression

Binomial logistic regression is a form of regression, which is used when the response variable is dichotomous (0/1; the independent predictor variables can be of any type). It is used very extensively in the medical, biological, and social sciences [37-41]. Logistic regression analysis can be used for any dichotomous response; for example, whether or not disease or death occurs. Any outcome can be considered dichotomous by distinguishing values that lie above or below a particular threshold. Depending on the context these may be thought of qualitatively as "favourable" or "unfavourable" outcomes. Logistic regression entails calculating the probability of an event occurring, given the values of various predictors. The logistic regression analysis determines the importance of each predictor in influencing the particular outcome. In SaSAT, we calculate the coefficients (*β*_*i*_) of the generalized linear model that uses the logit link function,

$$\begin{array}{cc} \text{logit}\left(p_{i}\right)=\ln\left(\frac{p_{i}}{1-p_{i}}\right)=\beta_{0}+\beta_{1}X_{1,i}+\beta_{2}X_{2,i}+\ldots +\beta_{m}X_{m,i}, & i=1\ldots n. \end{array}$$

where *p*_*i *_= *E*(*Y*|*X*_*i*_) = Pr(*Y*_*i *_= 1) and the *X*'s are the covariates; the solution for the coefficients is determined by maximizing the conditional log-likelihood of the model given the data. We also calculate the odds ratio (with 95% confidence interval) and p-value associated with the odds ratio.

There is no precise way to calculate *R*^2 ^for logistic regression models. A number of methods are used to calculate a pseudo-*R*^2^, but there is no consensus on which method is best. In SaSAT, *R*^2 ^is calculated by performing bivariate regression on the observed dependent and predicted values [42].

#### Sensitivity analyses for binary outputs: Kolmogorov-Smirnov

Like binomial logistic regression, the Smirnov two-sample test (two-sided version) [43-46] can also be used when the response variable is dichotomous or upon dividing a continuous or multiple discrete response into two categories. Each model simulation is classified according to the specification of the 'acceptable' model behaviour; simulations are allocated to either set *A *if the model output lies within the specified constraints, and set to *A*' otherwise. The Smirnov two-sample test is performed for each predictor variable independently, analysing the maximum distance *d*_max _between the cumulative distributions of the specific predictor variables in the *A *and *A*' sets. The test statistic is *d*_max_, the maximum distance between the two cumulative distribution functions, and is used to test the null hypothesis that the distribution functions of the populations from which the samples have been drawn are identical. P-values for the test statistics are calculated by permutation of the exact distribution whenever possible [46-48]. The smaller the p-value (or equivalently the larger *d*_max_(*x*_*i*_), the more important is the predictor variable, *X*_*i*_, in driving the behaviour of the model.

## Overview of software

SaSAT has been designed to offer users an easy to use package containing all the statistical analysis tools described above. They have been brought together under a simple and accessible graphical user interface (GUI). The GUI and functionality was designed and programmed using MATLAB^® ^(version 7.4.0.287, Mathworks, MA, USA), and makes use of MATLAB^®^'s native functions. However, the user is not required to have any programming knowledge or even experience with MATLAB^® ^as SaSAT stands alone as an independent software package compiled as an executable. SaSAT is able to read and write MS-Excel and/or MATLAB^® ^'*.mat' files, and can convert between them, but it is not requisite to own either Excel or Matlab.

**Figure 3:**
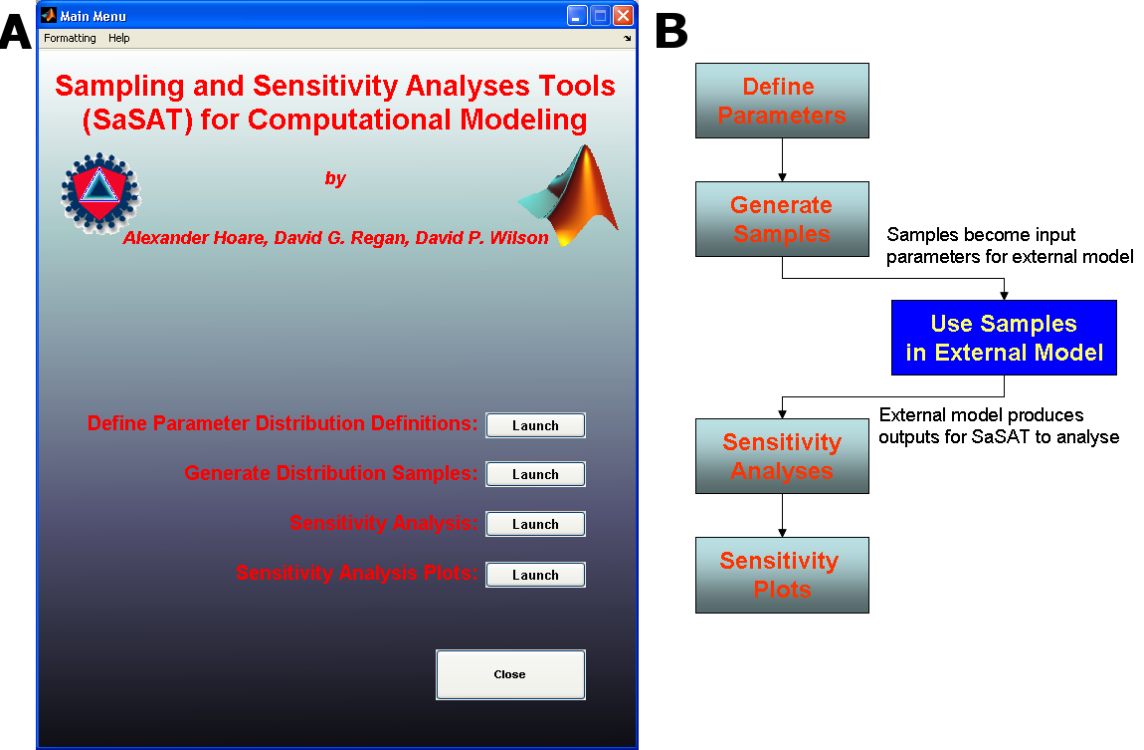
**(a) **The main menu of SaSAT, showing options to enter the four utilities; **(b) **a flow chart describing the typical process of a modelling exercise when using SaSAT with an external computational model, beginning with the user assigning parameter definitions for each parameter used by their model via the SaSAT '*Define Parameter Distribution*' utility. This is followed by using the '*Generate Distribution Samples*' utility to generate samples for each parameter, the user then employs these samples in their external computational model. Finally the user can analyse the results generated by their computational model, using the '*Sensitivity Analysis' *and '*Sensitivity Analysis Plots' *utility.

The opening screen presents the main menu (Figure 3a), which acts as a hub from which each of four modules can be accessed. SaSAT's User Guide [see Additional file 2] is available via the Help tab at the top of the window, enabling quick access to helpful guides on the various utilities. A typical process in a computational modelling exercise would entail the sequence of steps shown in Figure 3b. The model (input) parameter sets generated in steps 1 and 2 are used to externally simulate the model (step 3). The output from the external model, along with the input values, will then be brought back to SaSAT for sensitivity analyses (steps 4 and 5).

### Define parameter distributions

**Figure 4:**
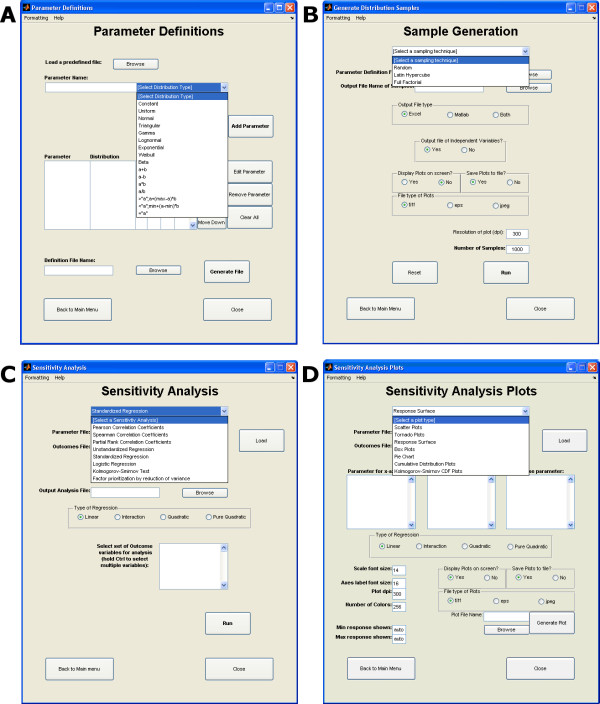
Screenshots of each of SaSAT's four different utilities: **(a) **The *Define Parameter Distribution Definition *utility, showing all of the different types of distributions available, **(b) **The *Generate Distribution Samples *utility, displaying the different types of sampling techniques in the drop down menu, **(c) **the *Sensitivity Analyses *utility, showing all the sensitivity analyses that the user is able to perform, **(d) **the *Sensitivity Analysis Plots *utility showing each of the seven different plot types.

The '*Define Parameter Distribution*' utility (interface shown in Figure 4a) allows users to assign various distribution functions to their model parameters. SaSAT provides sixteen distributions, nine basic distributions: 1) Constant, 2) Uniform, 3) Normal, 4) Triangular, 5) Gamma, 6) Lognormal, 7) Exponential, 8) Weibull, and 9) Beta; and seven additional distributions have also been included, which allow dependencies upon previously defined parameters. When data is available to inform the choice of distribution, the parameter assignment is easily made. However, in the absence of data to inform on the distribution for a given parameter, we recommend using either a uniform distribution or a triangular distribution peaked at the median and relatively broad range between the minimum and maximum values as guided by literature or expert opinion. When all parameters have been defined, a definition file can be saved for later use (such as sample generation).

### Generate distribution samples

Typically, the next step after defining parameter distributions is to generate samples from those distributions. This is easily achieved using the '*Generate Distribution Samples*' utility (interface shown in Figure 4b). Three different sampling techniques are offered: 1) Random, 2) Latin Hypercube, and 3) Full Factorial, from which the user can choose. Once a distribution method has been selected, the user need only select the definition file (created in the previous step using the '*Define Parameter Distribution*' utility), the destination file for the samples to be stored, and the number of samples desired, and a parameter samples file will be generated. There are several options available, such as viewing and saving a plot of each parameter's distribution. Once a samples file is created, the user may then proceed to producing results from their external model using the samples file as an input for setting the parameter values.

### Sensitivity analyses

The '*Sensitivity Analysis Utility' *(interface shown in Figure 4c) provides a suite of powerful sensitivity analysis tools for calculating: 1) Pearson Correlation Coefficients, 2) Spearman Correlation Coefficients, 3) Partial Rank Correlation Coefficients, 4) Unstandardized Regression, 5) Standardized Regression, 6) Logistic Regression, 7) Kolmogorov-Smirnov test, and 8) Factor Prioritization by Reduction of Variance. The results of these analyses can be shown directly on the screen, or saved to a file for later inspection allowing users to identify key relationships between parameters and outcome variables.

### Sensitivity analyses plots

The last utility, '*Sensitivity Analyses Plots' *(interface shown in Figure 4d) offers users the ability to visually display some results from the sensitivity analyses. Users can create: 1) Scatter plots, 2) Tornado plots, 3) Response surface plots, 4) Box plots, 5) Pie charts, 6) Cumulative distribution plots, 7) Kolmogorov-Smirnov CDF plots. Options are provided for altering many properties of figures (e.g., font sizes, image resolution, etc.). The user is also provided the option to save each plot as either a *.tiff, *.eps, or *.jpeg file, in order to produce images of suitable quality for publication.

## A simple epidemiological example

To illustrate the usefulness of SaSAT, we apply it to a simple theoretical model of disease transmission with intervention. In the earliest stages of an emerging respiratory epidemic, such as SARS or avian influenza, the number of infected people is likely to rise quickly (exponentially) and if the disease sequelae of the infections are very serious, health officials will attempt intervention strategies, such as isolating infected individuals, to reduce further transmission. We present a 'time-delay' mathematical model for such an epidemic. In this model, the disease has an incubation period of *τ*_1 _days in which the infection is asymptomatic and non-transmissible. Following the incubation period, infected people are infectious for a period of *τ*_2 _days, after which they are no longer infectious (either due to recovery from infection or death). During the infectious period an infected person may be admitted to a health care facility for isolation and is therefore removed from the cohort of infectious people. We assume that the rate of colonization of infection is dependent on the number of current infectious people *I*(*t*), and the infectivity rate *λ *(*λ *is a function of the number of susceptible people that each infectious person is in contact with on average each day, the duration of time over which the contact is established, and the probability of transmission over that contact time). Under these conditions, the rate of entry of people into the incubation stage is *λ I *(known as the force of infection); we assume that susceptible people are not in limited supply in the early stages of the epidemic. In this model *λ *is the average number of new infections per infectious person per day. We model the change between disease stages as a step-wise rate, i.e., after exactly *τ*_1 _days of incubation individuals become infectious and are then removed from the system after an infectious period of a further *τ*_2 _days. If 1/*γ *is the average time from the onset of infectiousness until isolation, then the rate of change in the number of infectious people at time *t *is given by

$$\frac{dI}{dt}=\lambda I\left(t-\tau_{1}\right)-\lambda e^{-\gamma \tau_{2}}I\left(t-\tau_{1}-\tau_{2}\right)-\gamma I\left(t\right).$$

**Figure 5:**
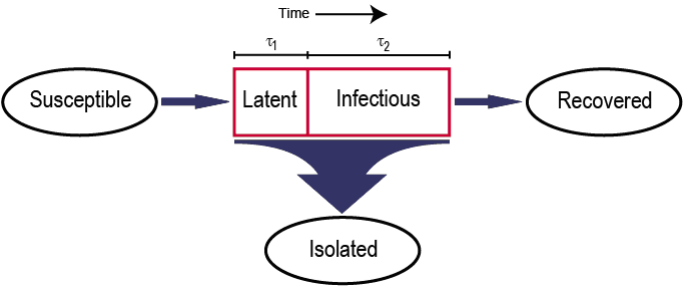
Schematic diagram of the framework of our illustrative theoretical epidemic model.

The exponential term arises from the fact that infected people are removed at a rate *γ *over *τ*_2 _days [49]. See Figure 5 for a schematic diagram of the model structure. Mathematical stability and threshold analyses (not shown) reveal that the critical threshold for controlling the epidemic is

$$R_{0}=\left(1-e^{-\gamma \tau_{2}}\right)\lambda /\gamma .$$

This threshold parameter, known as the basic reproduction number [50], is independent of *τ*_1 _(the incubation period). But at the beginning of the epidemic, if there is no removal of infectious people before natural removal by recovery or death (that is, if *γ *= 0), the threshold parameter becomes *R*_0 _= *λτ*_2_. If the infectious period (*τ*_2_) is long and there is significant removal of infectious people (*γ *> 0), then the threshold criterion reduces to *R*_0 _= *λ */*γ*. Intuitively, both of these limiting cases represent the average number of days that someone is infectious multiplied by the average number of people to whom they will transmit infection per day.

In order to contain (and effectively eliminate) an epidemic an intervention could involve attempting to quarantine infected individuals sufficiently quickly such that *γ *> *λ*. The sooner such an intervention is implemented, the greater the number of new infections that will be prevented. Therefore, an appropriate outcome indicator of the effectiveness of such an intervention strategy is the cumulative total number of infections over the entire course of the epidemic, which we denote as the 'attack number'. This quantity is calculated numerically from computer simulation. To investigate various interventions of quarantining, we model the steady increase from no isolation to a maximum of *p*% of infectious people that are isolated after an average of *τ*_3 _days of symptoms and this level of quarantining is maintained after *T *days. That is, $\gamma \left(t\right)=\{\begin{array}{cc} pt/\left(\tau_{3}T\right), & t<T\\ p/\tau_{3}, & t\geq T \end{array},$, where *t *is the time from the beginning of infectiousness of the first infected person (infectiousness could relate to the onset of symptoms, but not necessarily). Then, provided that *p*/*τ*_3 _> *λ *and this quarantine level is sustained, the epidemic will be eradicated.

There are three biological parameters that influence disease dynamics: *λ*, *τ*_1_, and *τ*_2 _(of which *λ *and *τ*_2 _are crucial for establishing the epidemic); and there are three intervention parameters crucial for eliminating the epidemic (*p*, *τ*_3_) and for reducing its epidemiological impact (*T*). In order to demonstrate this theoretical model and the tools of SaSAT we choose a hypothetical newly emergent disease with an incubation period of *τ*_1 _~ Γ (9,1/3) which specifies an average of 3 days and standard deviation of 1 day according to a Gamma distribution (Γ); an infectious period of *τ*_2 _~ *U*(6,10) days (i.e., a uniform distribution over the interval 6–10 days); and a transmission rate of *λ *~ *N*(0.4,0.1) new transmissions per day per infectious person (this is a Normal distribution with mean 0.4 and variance 0.1). This translates to an initial *R*_0 _prior to interventions of *R*_0 _~ *U*(6,10) × *N*(0.4,0.1) (which has a mean of 3.2 and standard deviation of ~0.93). We also investigate a range of different intervention strategies: isolating (1) 50%, (2) 75%, or (3) 95% of infectious people after an average of (a) 1 day, (b) 2 days, or (c) 3 days of symptoms, and scaling up the intervention to reach the maximal attainable level after either (i) 1 month, (ii) 2 months, or (iii) 3 months. This leads to a total of 27 intervention strategies.

To simulate the epidemics, samples are required from each of the three biological parameters' distributions. SaSAT's 'Define Parameter Distribution Definitions' utility allows these distributions to be defined simply. Then, SaSAT's 'Generate Distribution Samples' utility provides the choice of random, Latin Hypercube, or full-factorial sampling. Of these, Latin Hypercube Sampling is the most efficient sampling method over the parameter space and we recommend this method for most models. We employed this method here, taking 1000 samples, using the defined parameter file. Independent of SaSAT, this set of 1000 parameter values was used to carry out numerical simulations of the time-courses of the epidemic, and in each case we commenced the epidemic by introducing one infectious person. This was then carried out for each of the 27 interventions (a total of 27,000 simulations). For each simulation the time to eradicate the epidemic and the attack number were recorded. These variables became the main outcome variables used for the sensitivity analyses against the input parameters generated by the Latin Hypercube Sampling procedure.

**Figure 6:**
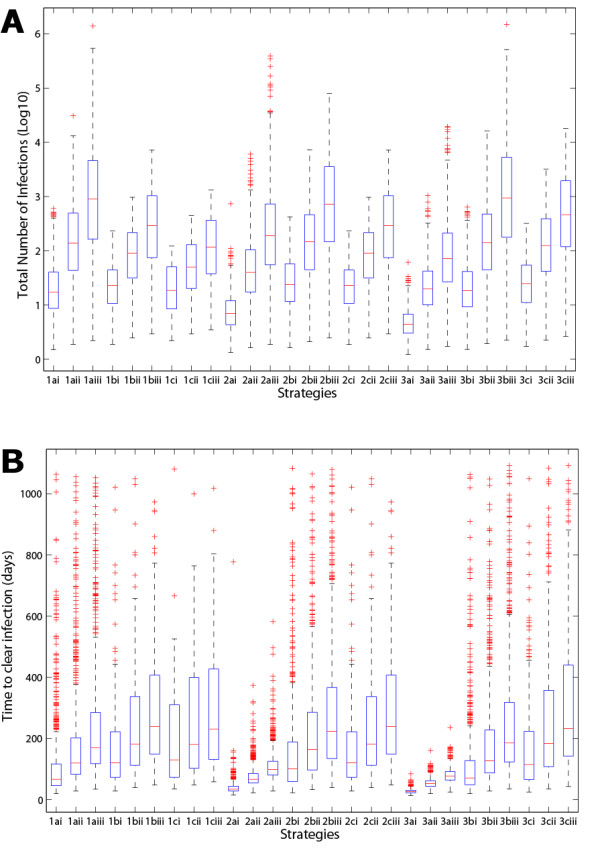
Box plots comparing the 27 different strategies used in the example model, with the whisker length set at 1.5 multiplied by the inter-quartile range, and red '+' showing the outliers: **(a) **the total number of infections caused by each strategy on a log10 scale, **(b) **the total time to clear the infection for each strategy.

**Figure 7:**
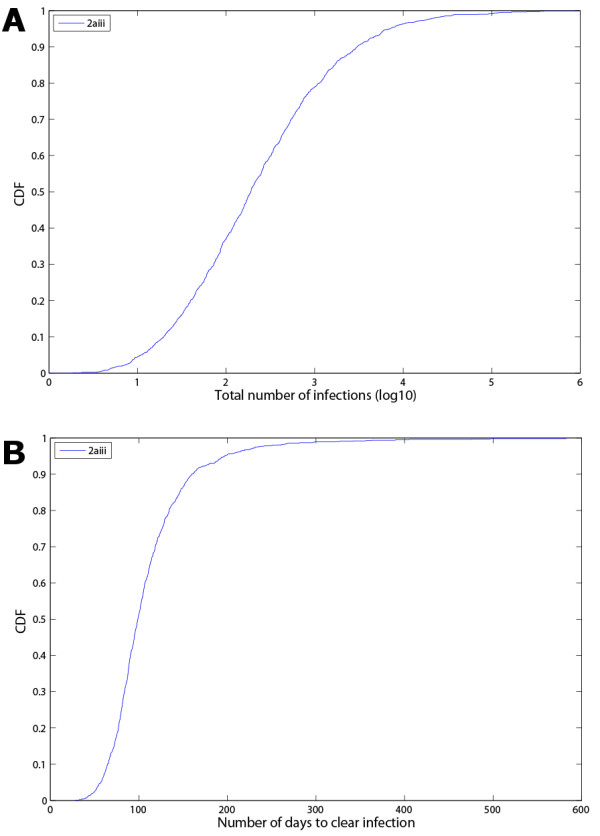
Cumulative density functions of: **(a) **the total number of infections (log10); and **(b) **the number of days to clear the infection, for a single strategy (2aiii: isolating 75% of infectious people after 1 day of infectiousness and achieving this level of intervention after 3 months).

**Figure 8:**
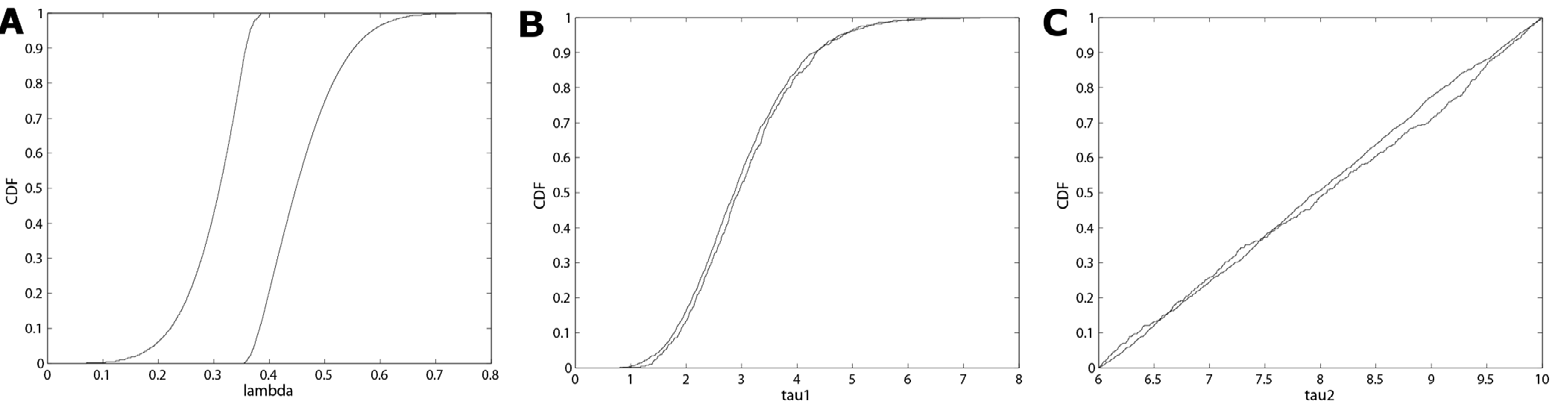
Kolmogorov-Smirnov plots of each parameter displaying the CDFs with the greatest difference between parameter subsets contributing to a 'success' or 'failure' outcome for each parameter (see Table 1): **(a) ***λ*, showing the largest maximum difference between the two CDFs, **(b) ***τ*_1_, showing very little difference, and **(c) ***τ*_2_, showing little difference similar to *τ*_1_. In this example a 'success' is defined as the total number of infections less than 100 at the end of the epidemic.

A research paper that is specifically focused on a particular disease and the impact of different strategies would present various figures (like Figure 6, generated from SaSAT's 'Sensitivity Analysis Plots' utility) and discussion around their comparison. However, for the purposes of this paper in demonstrating SaSAT we chose just one strategy (namely, 2aiii: attaining isolation of 75% of infectious individuals 1 day after symptoms begin, after 3 months from the commencement of the epidemic). The cumulative distribution functions of the distributions of time to eradicate the epidemic and the attack number were produced by SaSAT's 'Sensitivity Plots' utility and shown in Figs. 7a,b. The time until the epidemic was eradicated ranged from 28 to 583 days (99 median, IQR 81–126), and the total number of infections ranged from 2 to 501,263 (190 median, IQR 55–732). For the sake of illustration, if the goal of the intervention was to reduce the number of infections to less than 100, the importance of parameters in contributing to either less than, or greater than, 100 infections can be analysed with SaSAT by categorising each parameter set as a dichotomous variable. Logistic regression and the Smirnov test were used, within SaSAT's 'Sensitivity Analysis' utility and the results are shown in Table 1. As far as we are aware these methodologies have not previously been used to analyse the results of theoretical epidemic models. It is seen from Table 1 that *λ *(the infectivity rate) was the most important parameter contributing to whether the goal was achieved or not, followed by *τ*_2 _(infectious period), and then *τ*_1 _(incubation period). These results can be most clearly demonstrated graphically by Kolmogorov-Smirnov CDF plots (Fig. 8).

**Table 1:** Results of dichotomous variable sensitivity analysis: listing of the most important parameters in determining whether or not less than 100 people are infected by the epidemic (as determined by logistic regression and the Kolmogorov-Smirnov test).

**Parameter**	**Logistic Regression (R**^**2 **^**= 0.95)**	**Kolmogorov-Smirnov**
infectivity rate (*λ*)	*p *< 0.0001 odds > 10^8^	*p *< 0.0001 maxd = 0.93
incubation period (*τ*_1_)	*p *< 0.0001 odds 3.12 (2.34, 4.17)	*p *= 0.57 maxd = 0.05
infectious period (*τ*_2_)	*p *= 0.04 odds 1.29 (1.01, 1.64)	*p *= 0.20 maxd = 0.07

**Figure 9:**
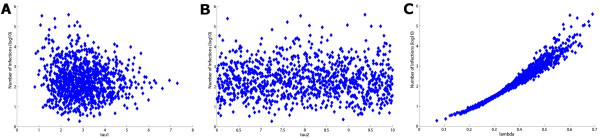
Scatter plots comparing the total number of infections (log10 scale) against each parameter: **(a) ***τ*_1_, shows some weak correlation, **(b) ***τ*_2_, shows little or no correlation, and **(c) ***λ*, showing a strong correlation (see Table 2 for correlation coefficients).

**Figure 10:**
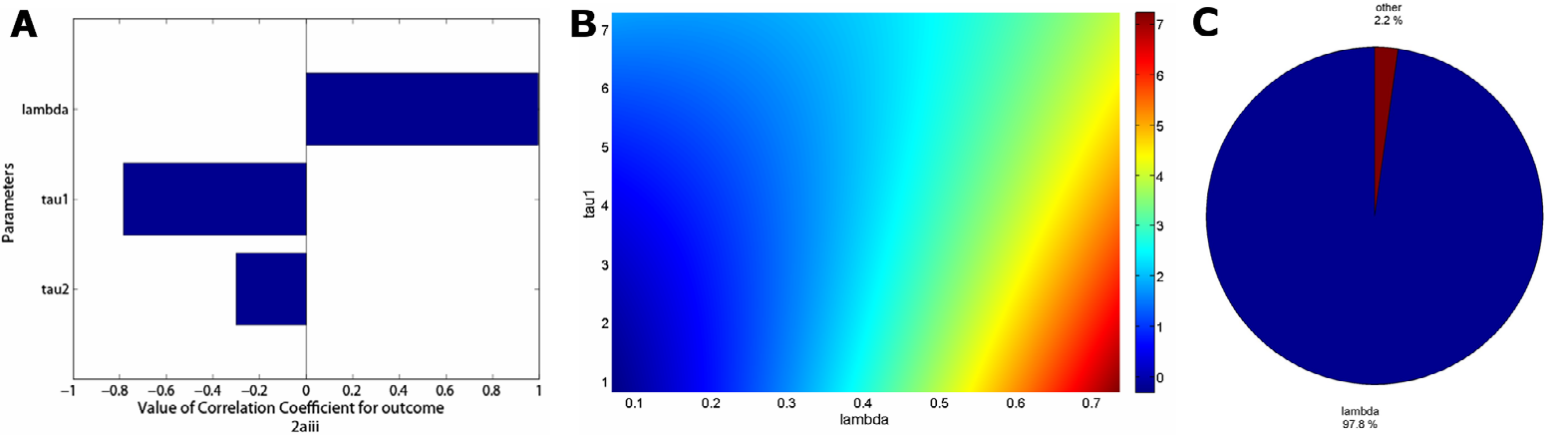
**(a) **Tornado plot of partial rank correlation coefficients, indicating the importance of each parameter's uncertainty in contributing to the variability in the time to eradicate infection. **(b) **Quadratic response surface (*R*^2 ^= 0.997, indicating extremely strong fit) of the total number of infections over the duration of the epidemic (on log10 scale) as it depends on *τ*_1 _(the incubation period) and *λ *(the infectivity rate). **(c) **Pie chart of factor prioritization sensitivity indices; this visual representation clearly shows the dominance of the infectivity rate for this model. Note that *τ*_1 _and *τ*_2 _have been combined under the title of 'other', this is because the sensitivity indices of these parameters are both relatively small in magnitude.

We investigated the existence of any non-monotonic relationships between the attack number and each of the input parameters through SaSAT's 'Sensitivity Plots' utility (e.g. see Figure 9); no non-monotonic relationships were found, and a clear increasing trend was observed for the attack number versus *λ*, the infectivity rate. Then, it was determined which parameters most influenced the attack number and by how much. To conduct this analysis, SaSAT's 'Sensitivity Analyses' utility was used. The calculation of PRCCs was conducted; these are useful for ranking the importance of parameter-output correlations. Another method that we implemented for ranking was the calculation of standardized regression coefficients; the advantage of these coefficients is the ease of their interpretation in how a change in one parameter can be offset by an appropriate change in another parameter. A third method for ranking the importance of parameters, not previously used in analysis of theoretical epidemic models as far as we are aware, is factor prioritization by reduction of variance. These indicators of importance of parameters provided consistent rankings, as shown in Table 2; we calculated these indices for linear, interaction, pure quadratic and full quadratic response hypersurfaces and they were all in very close agreement (all indices were equivalent to at least 2 decimal places for each statistical model and so we show results just for the full quadratic case (R^2 ^= 0.997)). The rankings for all correlation coefficients can also be shown as a tornado plot (see Figure 10a).

**Table 2:** Results of sensitivity analysis: impact of the variability in the input variables in influencing variability in the attack number (total cumulative number of infected people), as determined by (i) partial rank correlation coefficients, (ii) standardized regression coefficients, and (iii) factor prioritization by reduction of variance.

**Parameter**	**Partial rank correlation coefficient**	**Standardized regression coefficient**	**Sensitivity index (reduction of variance)**
infectivity rate (*λ*)	0.995	0.982	97.7%
incubation period (*τ*_1_)	-0.783	-0.146	2.1%
infectious period (*τ*_2_)	-0.300	-0.025	0.2%

The influence of combinations of parameters on outcome variables can be presented visually. Response surface methodology is a powerful approach for investigating the simultaneous influence of multiple parameters on an outcome variable by illustrating (i) how the outcome variable will be affected by a change in parameter values; and (ii) how one parameter must change to offset a change in a second parameter. Figure 10b, from SaSAT's 'Sensitivity Plots' utility shows the pairings of the impact of infectivity rate (*λ*) and the incubation period (*τ*_1_) on the attack number. Factor prioritization by reduction of variance is a very useful and interpretable measure for sensitivity; it can be represented visually through a pie-chart for example (Fig. 10c).

## Conclusion

In this paper we outlined the purpose and the importance of conducting rigorous uncertainty and sensitivity analyses in mathematical and computational modelling. We then presented SaSAT, a user-friendly software package for performing these analyses, and exemplified its use by investigating the impact of strategic interventions in the context of a simple theoretical model of an emergent epidemic.

The various tools provided with SaSAT were used to determine the importance of the three biological parameters (infectivity rate, incubation period and infectious period) in (i) determining whether or not less than 100 people will be infected during the epidemic, and (ii) contributing to the variability in the overall attack number. The various graphical options of SaSAT are demonstrated including: box plots to illustrate the results of the uncertainty analysis; scatter plots for assessing the relationships (including monotonicity) of response variables with respect to input parameters; CDF and tornado plots; and response surfaces for illustrating the results of sensitivity analyses.

The results of the example analyses presented here are for a theoretical model and have no specific "real world" relevance. However, they do illustrate that even for a simple model of only three key parameters, the uncertainty and sensitivity analyses provide clear insights, which may not be intuitively obvious, regarding the relative importance of the parameters and the most effective intervention strategies.

We have highlighted the importance of uncertainty and sensitivity analyses and exemplified this with a relatively simple theoretical model and noted that such analyses are considerably more important for complex models; uncertainty and sensitivity analyses should be considered an essential element of the modelling process regardless of the level of complexity or scientific discipline. Finally, while uncertainty and sensitivity analyses provide an effective means of assessing a model's "trustworthiness", their interpretation assumes model validity which must be determined separately. There are many approaches to model validation but a discussion of this is beyond the scope of the present paper. Here, with the provision of the easy-to-use SaSAT software, modelling practitioners should be enabled to carry out important uncertainty and sensitivity analyses much more extensively.

## Appendix

### SaSAT

Sampling and Sensitivity Analysis Tools.

### Sampling

Selection of values from a statistical distribution defined with a probability density function for a range of possible values. For example, a parameter (*α*) may be defined to have a probability density function of a Normal distribution with mean 10 and standard deviation 2. Sampling chooses *N *values from this distribution.

### Mathematical/Theoretical/Computational Model

A set of mathematical equations that attempt to describe a system. Typically, the model system of equations is solved numerically with computer simulations. Mathematical models are different to statistical models, which are usually described as a set of probability distributions or equation to fit empirical data.

### Input parameter/predictor/explanatory variable/factor

A constant or variable that must be supplied as input for a mathematical model to be able to generate output. For example, the diameter of a pipe would be an input parameter in a model looking at the flow of water.

### Outcome/Output/response variable

Data generated by the mathematical model in response to a set of supplied input parameters, usually relating to a specific aspect of the model, e.g., the amount of water flowing into a container from a pipe/s of a certain diameter.

### Uncertainty Analysis

Method used to assess the variability (prediction imprecision) in the outcome variables of a model that is due to the uncertainty in estimating the input values.

### Sensitivity Analysis

Method that extends uncertainty analysis by identifying which parameters are important in contributing to the prediction imprecision. It quantifies how changes in the values of input parameters alter the value of outcome variables. This allows input parameters to be ranked in order of importance, that is, the parameters that contribute the most to the variability in the outcome variable.

### LHS

Latin Hypercube Sampling. This is an efficient method for sampling multi-dimensional parameter space to generate inputs for a mathematical model to generate outputs and conduct uncertainty analysis.

### PRCC

Partial Rank Correlation Coefficient. A method of conducting sensitivity analysis.

### Monotonic

A relationship or function which preserves a given trend; specifically, the relationship between two factors does not change direction. That is, as one factor increases the other factor either always increases or always decreases, but does not change from increasing to decreasing.

### GUI

Graphical user interface.

## Authors' contributions

AH wrote the graphics user interface code for SaSAT, developed the software package, wrote code for functions implemented in SaSAT, wrote the User Guide, performed analyses with the example model, produced all figures, and contributed to the Outline of Software section. DR and DW contributed to the overall conceptualisation and design of the project, developed code for the uncertainty and sensitivity algorithms. DR contributed to preparation of the manuscript. DW designed the example model, prepared the manuscript, and supervised the software design.

## Supplementary Material

Additional file 1 Download SaSAT. Information for downloading SaSAT software. Click here for file

Additional file 2 User guide. User guide for SaSAT software package. Click here for file
