# Supplementary material for: Sampling and sensitivity analyses tools (SaSAT) for computational modelling
Source: Theor Biol Med Model. 2008 Feb 27;5:4. doi: 10.1186/1742-4682-5-4 (PMC2292159; doi:10.1186/1742-4682-5-4)
Supplement: Additional file 1 — Download SaSAT. Information for downloading SaSAT software. [file 1742-4682-5-4-S1.pdf]

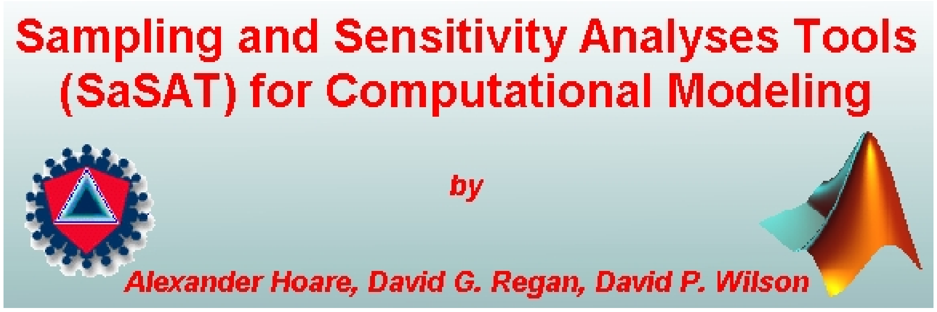

SaSAT software can be freely downloaded from the following website:

http://www.nchecr.unsw.edu.au/NCHECRweb.nsf/page/BioModInfectDis

Alternatively, information about downloading and installing SaSAT can be obtained by contacting the authors via email (dwilson@nchecr.unsw.edu.au). The authors can also distribute the software on a CD upon request.

Password

Upon installation a password is required. The password is sasat.

There are two files available for download: only one is required

SaSAT can be downloaded with or without MCR installer. The version with MCR installer is substantially larger! The MCR installer is required for users who do not have MATLAB installed on their machines.

<u>What is MCR installer?</u>

MCR stands for MATLAB Component Runtime. The MCR allows MATLAB compiled programs to run outside of MATLAB. It works in a similar way to the Java Virtual Machine (allows the execution of java scripts), and contains a series of *.dll and various other files, that are able to interpret and execute a program written in the MATLAB language. The MCR is specific to the version of MATLAB that produced it. For SaSAT, we used version 7.6 of the MCR and was written in MATLAB 7.4.0 (R2007a). If you do not have version 7.6 of the MCR installed, or are unsure, then it is best to download the SaSAT with the MCR. See: http://www.mathworks.com/access/helpdesk/help/toolbox/compiler/f12-999353.html for more information regarding the MCR.
