# Supplementary material for: Sampling and sensitivity analyses tools (SaSAT) for computational modelling
Source: Theor Biol Med Model. 2008 Feb 27;5:4. doi: 10.1186/1742-4682-5-4 (PMC2292159; doi:10.1186/1742-4682-5-4)
Supplement: Additional file 2 — User guide. User guide for SaSAT software package. [file 1742-4682-5-4-S2.pdf]

Sampling and Sensitivity Analysis Tools for Computational Modelling: User Guide

Alexander Hoare,

David G. Regan,

David P. Wilson

National Centre in HIV Epidemiology and Clinical Research, University of New South Wales, Sydney, Australia.

Contents

Chapter 1: Getting Started-Installation Guide: 3
Windows XP/Vista 32bit 3
Windows XP/Vista 64bit 3
Main Menu 4
Chapter 2: Define Parameter Distribution Definitions 6
Step-by-Step Guide: 8
Chapter 3: Generate Distribution Samples 10
Step-by-Step Guide: 11
Plot Description: 12
Chapter 4: Sensitivity Analysis 13
Step-by-Step Guide: 14
Displayed Data Information: 15
Chapter 5: Sensitivity Analysis Plots 21
Step-by-Step Guide: 22
Chapter 6: Other Tools 24
File Converter 24
Mat File Formatter 25
Chapter 7: Notes about file structure 26
Chapter 8: List of Error messages 28
Parameter Definitions: 28
Sample Generation: 28
Sensitivity Analysis 29
Sensitivity Plots 30
Chapter 9: Glossary 31

Chapter 1: Getting Started-Installation Guide:

Windows XP/Vista 32bit

1) Run the ‘SaSAT32_installer.exe’.
2) You will then be prompted to choose a location to unzip the SaSAT files, the default location in ‘C:\Program Files\SaSAT’. This will unzip several files, including ‘MCRInstaller.exe’ and ‘UserGuide.pdf’.
3) When it has finished uncompressing these files, it should automatically start the ‘MCRInstaller.exe’. ‘MCRInstaller’ installs the Matlab Component Runtime, which contains all of the necessary library files for the program to work; this is similar to the Java Virtual Machine. *It is advised that you install this to the default location*.
4) Once the MCR has been installed, in order to run SaSAT the SaSAT.exe file should be executed. This will bring up a command window, and begin to unpack a ‘*.ctf’ file. Once it has finished uncompressing the files, it will run SaSAT. Note, it will only need to unpack the *.ctf file once. For future use of SaSAT this procedure is simplified and SaSAT will load quickly.
5) You may now run SaSAT any time you wish.
6) To run SaSAT in the future, use the SaSAT.exe file; we recommend creating a shortcut to this file on your desktop.

Windows XP/Vista 64bit

Similar to the 32bit install, only you must use the 64bit version of the program. Follow the same steps as above but replacing ‘SaSAT32_pkg.exe’ with ‘SaSAT64_pkg.exe’.

Main Menu

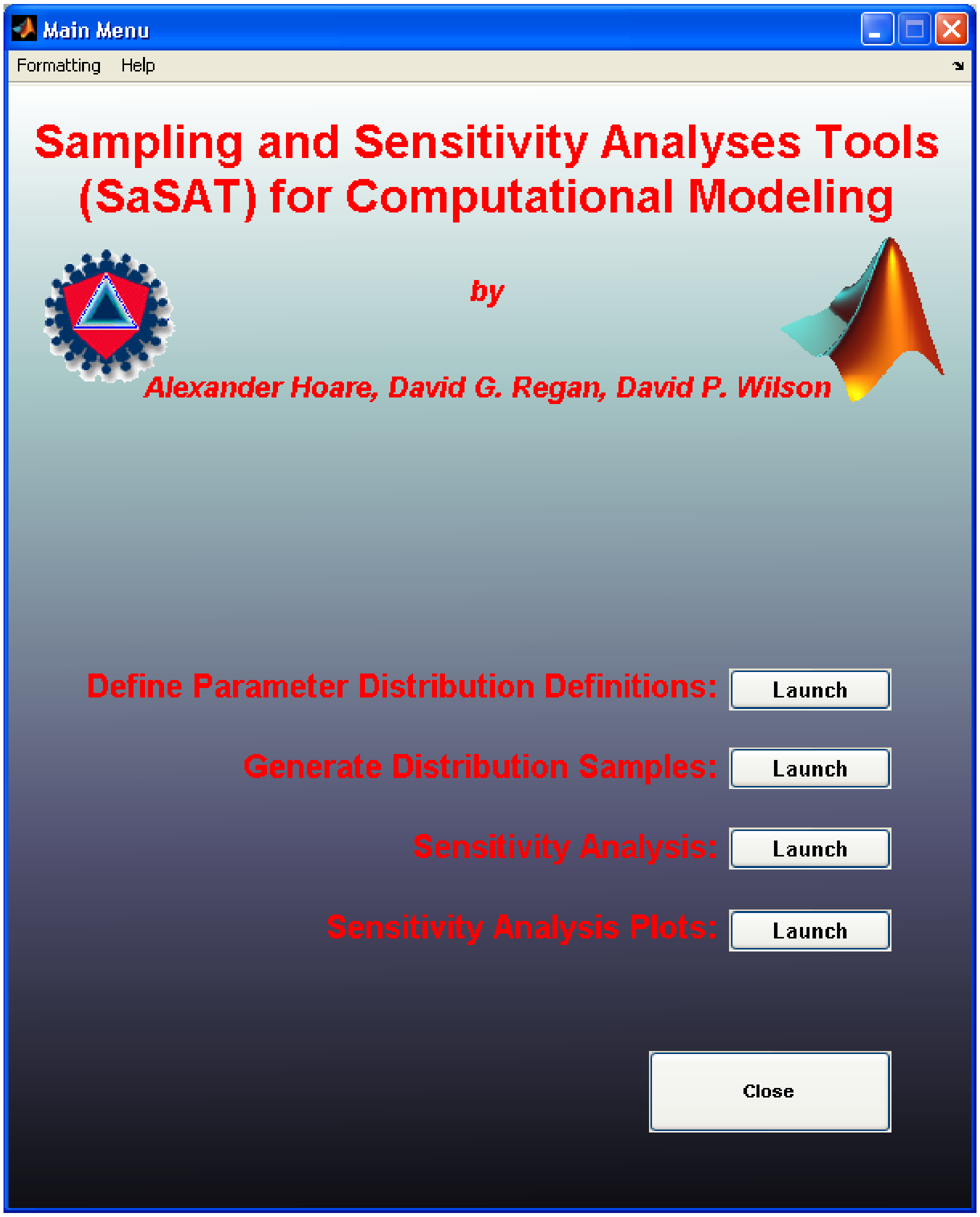

Figure 1: Main Menu, access to utilities are via the buttons on the right, and additional formatting tools can be accessed via the menu bar.

The 'Main Menu' (shown in Figure 1) is the initial screen that users will see. It links all four utilities of the SaSAT package together. These are:

Define Parameter Distribution Definitions – takes you through to Definitions page that will allow you to assign your parameter distributions (see Chapter 2).

Generate Distribution Samples – this utility will interpret your parameter definitions and create a parameter samples file for use in your model (see Chapter 3).

Sensitivity Analysis – this utility allows you to preform a range of sensitivity analysis tests on the data generated from your model (see Chapter 4).

Sensitivity Analysis Plots – here you will be able to make plots from your output data (see Chapter 5).

Typically, you would start with the Define Parameter Distribution utility to assign each parameter a distribution function. The next step will then involve using the Generate Distribution Samples utility, which will create samples from the distributions assigned to each parameter. At this stage you would then use these created samples in your model and collect a series of outcome variables. With these results, you can then use the Sensitivity Analysis utility to examine your results.

Chapter 2: Define Parameter Distribution Definitions

The *Define Parameter Distribution Definitions* utility allows you to create a file that provides a definition for your parameters. The way your parameter is defined depends on the type of distribution assigned to it. In this package, we have provided 16 different types of distributions (shown in the Table below).

Distribution Type	Number of input arguments	Description
Constant	1	This will create a vector with a single value as entered by the user.
Uniform	2	A uniform sample will be taken between a minimum and maximum value, as entered by the user.
Normal	2	Input consists of a mean and standard deviation
Triangular	3	User provides the minimum, maximum and peak values of a triangle. The peak must lie between the min and max range.
Gamma	2	Users must define the shape parameter k and scale parameter θ.
Lognormal	2	Similar to the Normal distribution, users must enter a mean and standard deviation
Exponential	1	The distribution only requires users to enter a mean value.
Weibull	2	Users must define the shape parameter k, and scale parameter λ.
Beta	2	Users must define two shape parameters, α and β
a+b	2	This is the sum of parameter 'a' and parameter 'b'. Parameters 'a', and 'b', must be previously defined, and users simply enter the parameter names
a-b	2	The difference between parameters 'a' and 'b'
a*b	2	The product parameters of 'a' and 'b'
a/b	2	The quotient parameters of 'a' and 'b'
>a, a+(max-a)*b	3	This lets you set up a distribution that is always great than a previous parameter value. Parameter 'b' is a previously defined parameter that is usually uniformly distributed between 0 and 1. And 'max' is the maximum value of 'a'
<a, min + (a-min)*b	3	Similar to above, but this distribution ensures that values are less than that for parameter 'a'.
= a	1	Creates a parameter that has the same values as parameter 'a'

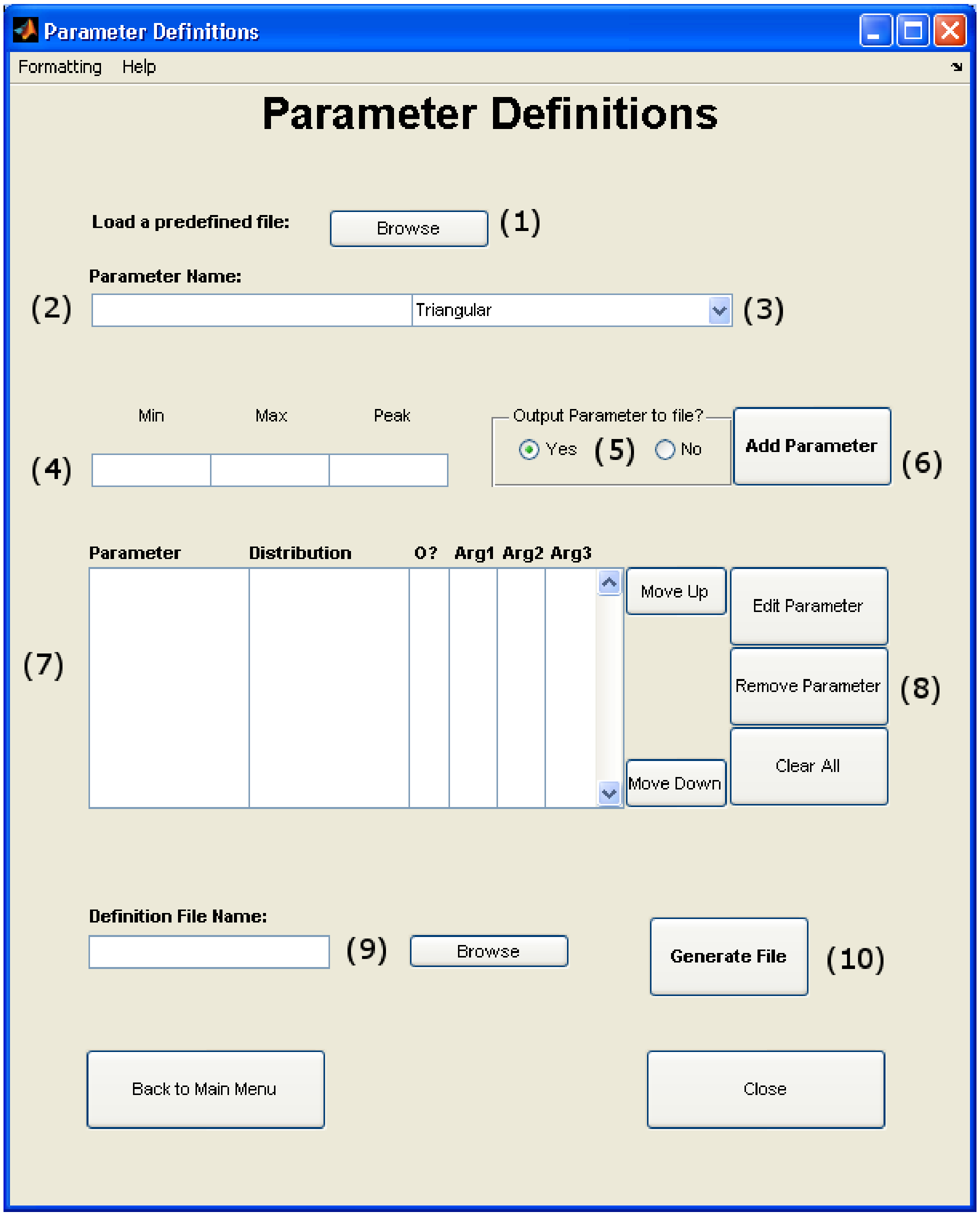

Figure 2: Screenshot of the Parameter definition utility. (1) Browse button for selecting a previous file (step 1), (2) Text field for parameter name (step 2), (3) drop down menu to select distribution type (step 3), (4) Text fields to assign arguments for the distribution (step 4), (5) Radio buttons to output the parameter (step 5), (6) Add Parameter button (step 6), (7) Listboxes that display defined parameters (step 7), (8) group of editing buttons (step 8), (9) Text field and Browse button (step 9), (10) Generate File button (step 10).

Step-by-Step Guide (refer to Figure 2):

1) If you have a previously created a file, you can press the 'Browse' button at the top of the screen. This will open up a file directory browser and you can select your previously saved definition file. Only Excel and MAT files are accepted as valid file types for input. Once you have selected your file, click the open button on the bottom right. The data will then be extracted from the file and displayed in the listboxes. This may take several moments depending on the type and size of the file. Once loaded, you are able to add, remove and edit definitions as before.
2) If you do not have a previously created file, you will need to start here. In the text field, enter the name of your parameter (if this is a new definition file, enter the first of potentially many parameter names).
3) Next, you must select what type of distribution you wish to assign to this parameter name. For a list of distributions see Table 1.
4) When a distribution has been selected, some text fields will be displayed underneath the parameter name field. Depending on the type of distribution selected there will be anywhere from 1 to 3 text fields; arguments associated with the distribution. Atop each text field, there will be a description of what needs to be inputted into the text field.
5) When you have given the appropriate values to the arguments, you must then decide whether you want this parameter to be outputted or not. Outputting the parameter means that it will be displayed in the parameter file created in the next chapter. You would usually not output the parameter if it is a 'dummy' parameter that will be used to define a dependent parameter at a later time and is itself not directly required for the computational model.
6) When you are happy with your choices for the definition of your parameter, you may press the 'Add Parameter' button. Note: if a mistake is made, you can edit the parameter at any time. See step 8a for more information about editing parameters.
7) When the 'Add Parameter' button is pressed, all of the details of the new parameter will be displayed in these listboxes. These series of steps can be repeated for all subsequent parameters that are to be defined. The newly defined parameters will appear at the bottom of the listboxes under all previously defined parameters.
8) **(Optional step required only for editing)** This group of buttons allows you to change the order of the parameters. The 'Move Up', and 'Move Down' buttons allow you to move the selected parameter up and down the list. This is helpful if you need to change the order of your parameters.
   a. The 'Move Up', and 'Move Down' buttons allow you to move the highlighted parameter up and down the list. This is handy if you need to change to the order of your
   b. The 'Edit Parameter' button allows you to edit the highlighted parameter. When pressed, the details of that parameter are automatically transferred into the appropriate fields, which you can then edit. When you have finished making the changes you want, press the 'Add Parameter' button to update the display. Important Note: once you have pressed the edit

button, you must not highlight any other parameter, or it will be overwritten when the add parameter button is pressed.

   c. The ‘Remove Parameter’ button allows you to remove the highlighted parameter from the list. A warning message will come up asking you to confirm the removal.
   d. The last button is the ‘Clear All’ button; this allows you to reset your current project. A warning message will be displayed asking you to confirm the reset. Be warned that all current definitions will be lost unless you have saved them to a file.

9) Once you have finished defining your parameters, the next step is to choose a file or filename to save your definitions. There are two way to pick a filename:
   i. The text box – here you may simply type in the filename. However, you must specify a file extension such as ‘filename.*xls*’, or ‘filename.*mat*’, otherwise an error will occur when generating the file.
   ii. The ‘Browse’ button – this allows you select a previously created file, or to choose the location of the file. If you are not going to overwrite a previous file, then just type in the filename, you must select either ‘*.xls’, or ‘*.mat’ from the drop down menu. If a previously defined file was opened (step 1) then the same file will automatically be selected to be updated at this step.

10) **Important:** Once you have chosen your file name, you can then click the ‘Generate File’ button. This will create the definition file at the specified location, if no location is given, it will create it in the same directory as the program. If you have chosen to output to an Excel file, this may take a few moments, however a message will be displayed upon completion.

Chapter 3: Generate Distribution Samples

Having created an appropriate parameter definition file, the next stage is to generate samples from the chosen distributions. We have provided three sampling schemes in which to sample from each distribution. These are: 1) Random sampling, 2) Latin Hypercube sampling, and 3) Full Factorial sampling. For most computational models we recommend Latin Hypercube sampling.

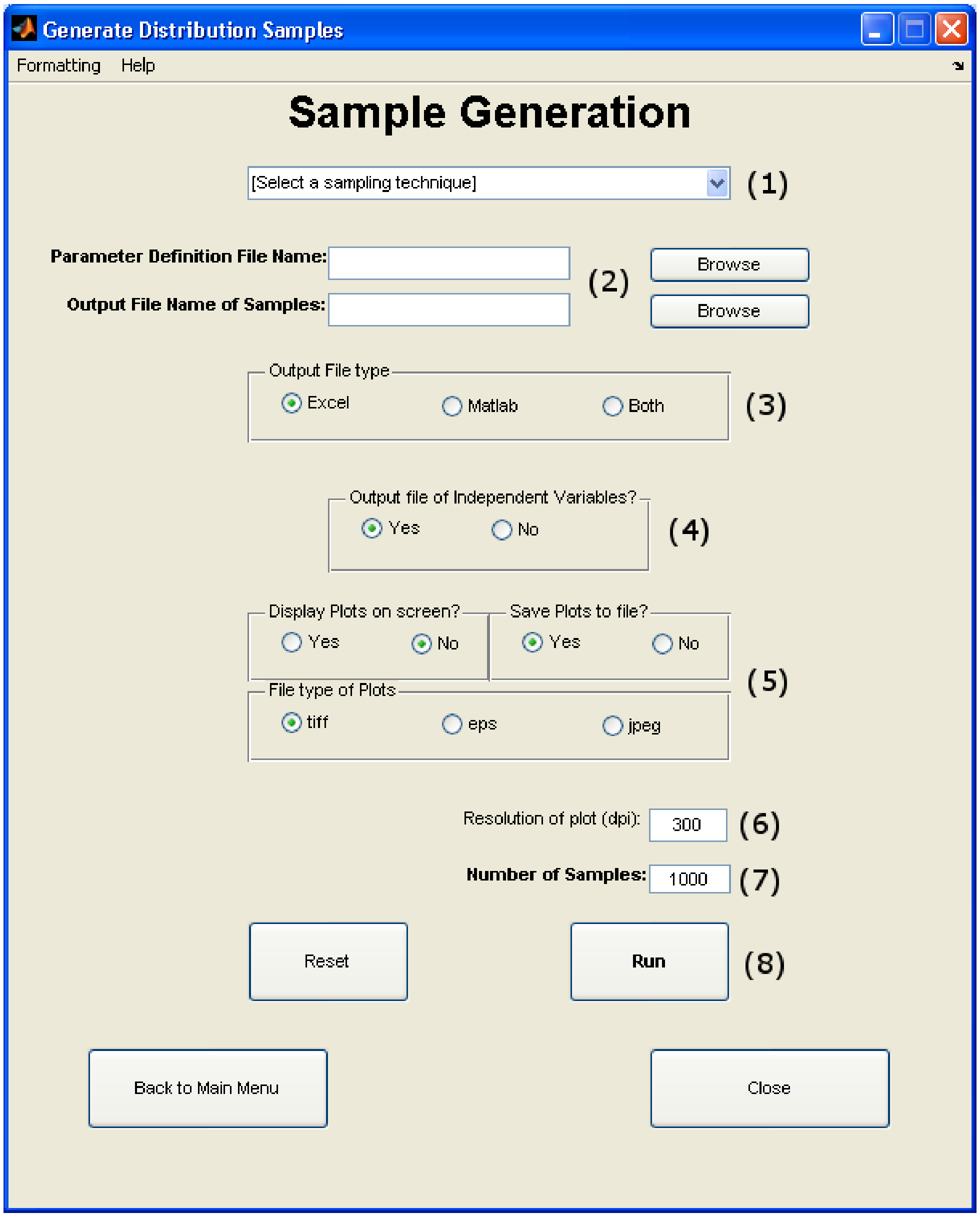

Figure 3: (1) Drop down menu (step 1), (2) file selection area (steps 2-3), (3) File type selection (step 4), (4) Create Independent Variable file (step 4), (5) Display plot and save plot options (steps 5-7), (6) Text field for plot resolution (step 8), (7) Text field to choose the number of samples (step 9), (8) Run and Reset buttons (step 10).

Step-by-Step Guide (refer to Figure 3):

1) Select the sampling technique that you wish to use from the drop down menu.
2) In the Parameter Definition File Name text box, you must enter the name of the definition file that you created in the previous chapter. If the file is in the same directory as the program, you can simply type in the name, making sure to include the file extension.
3) You must then choose a filename for the Parameter Sample file. Here it is not necessary to choose a file extension as you will have that option later (step 4). You can also use the 'Browse' button to select a previous file, or to choose the location of the file.
4) Next you select the file type that you wish to work with. The default type is Excel, but you are able to select .MAT, or to output both file types.
5) Your next step is to choose whether you wish to output a file of independent parameters. Choosing to output the file means that all parameters that are considered independent will be placed into a separate file, called Independent_parameters.* (where * is the same extension as chosen in step 4). This will help later on when you want to perform some sensitivity analysis on your results, as you will not need to edit your parameter file to exclude the dependent parameters.
6) In this next step, you have the option of choosing to view the plots of each distribution, and where the samples were taken from. If this option is selected a warning will popup when the 'Run' button is pushed. If you have a large number of parameters, it is advised that you do not display the plots to the screen, as this will put a significant load on your system.
7) If you still wish to see the plots, you may choose to save them to a file. This will create the image files in the same folder as the program. Again, your system will run slower than if you chose not to save the plots to a file. Note, if you choose to display the plots and save them to a file, wait until you receive the successful completion message before you close the figure plot windows.
8) If you choose to save the plots to a file, some new options will become visible. Here you may select the type of file you wish for your plots, we give you the option to output to *.tiff, *.eps, and *.jpeg. A text box also appears, this lets you set the dpi (dots per inch), and in effect the resolution of your files. We have set the default value to 300dpi, which produces plots with a resolution of 2402x1801 pixels (a value of 'x' dpi results in a resolution of $8x$+2 by $6x$+1 pixels).
9) The last thing to set before you can run the program is the number of samples that you wish to collect. We have set the default value to 1000 for the random and Latin hypercube sampling schemes; this will correspond to 1000 different sets of parameter values. For full factorial sampling, the number of different sets of parameter values is: (number of samples) to the power of (number of parameters). Full factorial sampling can result in a very large number of samples and we do not recommend it for large numbers of parameters or large numbers of samples; a warning message will appear before continuing.
10) The final step is for you to review your choices; should you wish to start again, you may press the 'Reset' button. This will reset everything back to its initial state. However, if you are happy with your choices, your may press the 'Run'

button to create the file. You will get a message acknowledging the success or failure of the action.

11) If successful, you are now ready to use your samples file for you model.

Plot Description:

As explained above, we have provided the option to view or save the plots of the parameter distributions. Here we provide a guide explaining the information displayed in the plots.

Figure 4 shows the plot of a normal distribution with 10 samples taken. The solid blue line traces out the curve representing the probability distribution function. The red vertical lines divide the curve into areas of equal probability. The blue crosses on the x-axis represent each sample used in the sample.

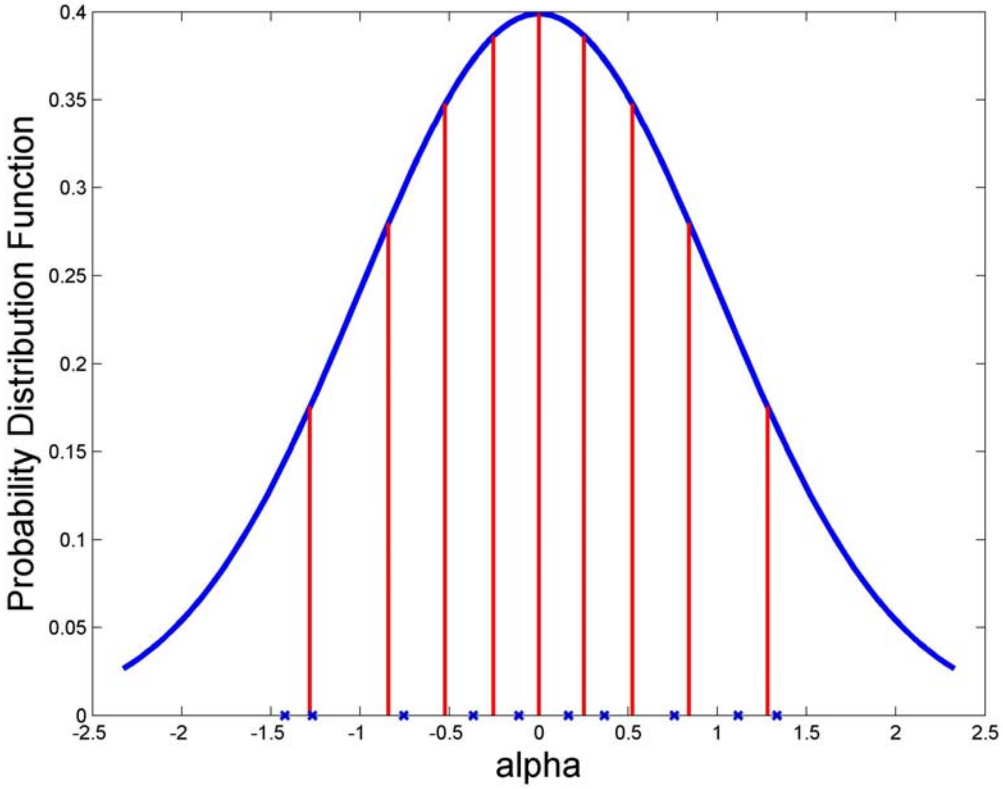

Figure 4: Example plot of a normal distribution with mean 0 and standard distribution 1. Blue curve indicates the distribution function, red vertical lines indicate the division of the curve into areas of equal probability, and blue 'x' represent the value of samples taken.

Chapter 4: Sensitivity Analysis

Once you have finished running your model using the samples generated in the '*Generate Distribution Samples*' utility, you will most likely have several output variables to analyse. In this portion of the GUI, several types of sensitivity analysis tools are provided, that should provide valuable insights into the relationship between your input parameters and your output variables.

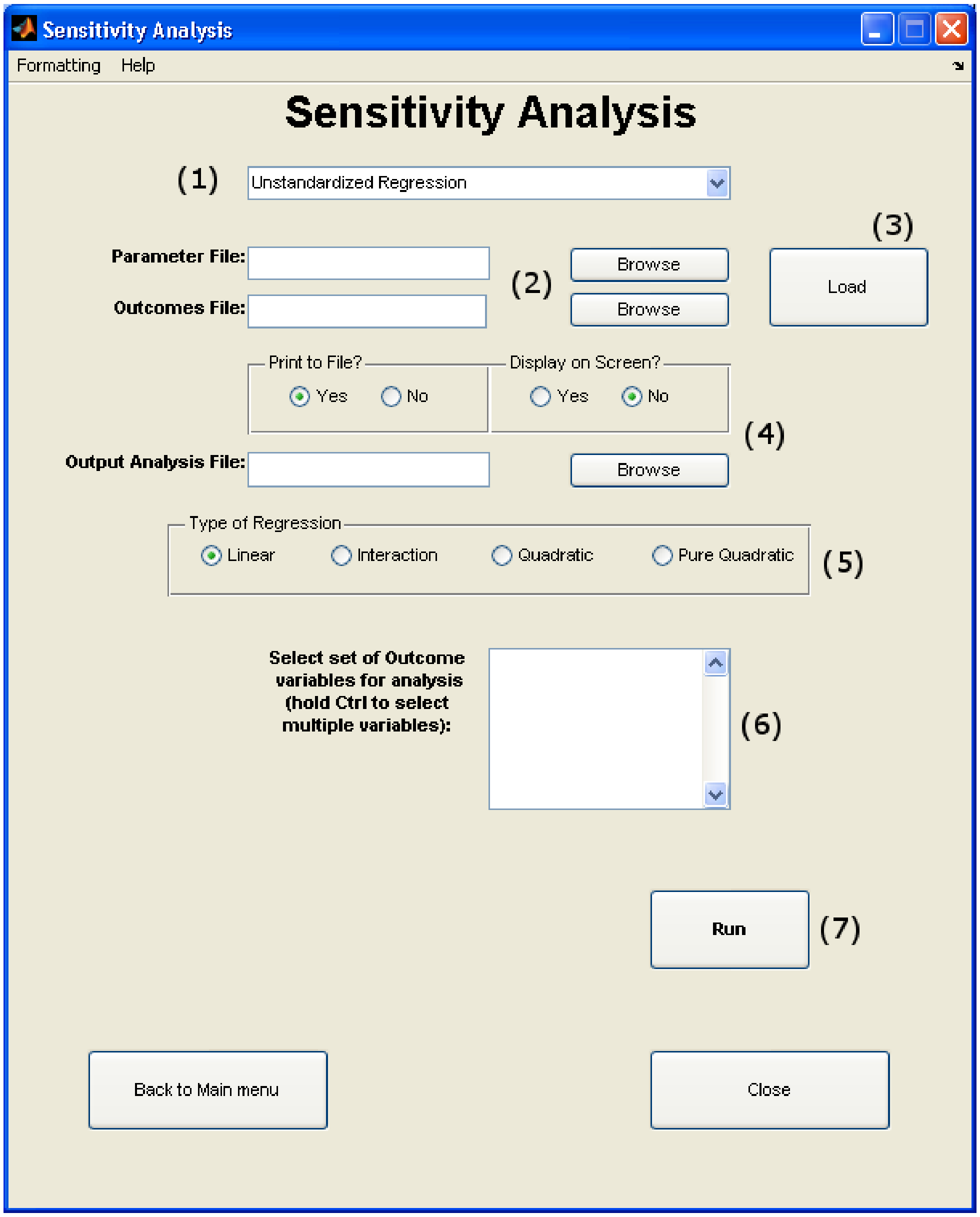

Figure 5: (1) Drop down menu (step 1), (2) Text fields and browse button (steps 2-3), (3) Load button (step 7), (4) File saving options (steps 4-5), (5) Radio buttons for regression type (step 6), (6) Listbox to select outcome variables (step 7), (7) Run button (step 8).

Step-by-Step Guide (refer to Figure 5):

1) First you must select the type of sensitivity analysis that you wish to perform. There are several options depending on what you wish to do. Also, each option has different sets of inputs required from the user. Some steps in this guide will not apply to all types of analyses.
2) All types of sensitivity analyses require you to input a parameter file. This file should be the same one created on the Sample Generation page. It is here that it would be a good idea to use the independent parameter file, as some of the sensitivity analyses provided will not work properly if there are parameters that are constant, or dependent on another parameter.
3) The next text field is for the file containing all of your output data. Each row in the output file should correspond to the same row of parameters in the parameter file. See Chapter 6 for examples on how to set up your files.
4) You have the option to print the sensitivity analysis data to a file, or alternatively you can display the data on screen. For Pearson, Spearman, and Partial Rank correlation coefficients, having Microsoft Office Spreadsheet 10.0 or 11.0, which are a part of Office Web Components (OWC), installed will provide an easier way to view the data. This comes free with Office 2003 and earlier versions. However, it is available to download from: (http://www.microsoft.com/downloads/details.aspx?FamilyID=7287252c-402e-4f72-97a5-e0fd290d4b76&DisplayLang=en). It is not necessary to have OWC installed, the program will use an alternative method to display the correlation coefficient information. This will occur automatically. For the other types of sensitivity analyses no other software is required. Note that if you choose not to print to a file, and to view the information on screen, you will still have the option to save to a file.
5) If you choose to print to a file, you will have another text field to fill out. This is to name the analysis file. You have the same options as with previous file naming areas, in that you can browse for another file or location. Note, if you select not to print to a file, this text field will not be visible.
6) If all you have chosen to do is a Pearson, Spearman, or Partial Rank test, then you are done, and can skip to step 8. However, if you have chosen to use ‘Unstandardized Regression’, ‘Standardized regression’, or ‘Factor Prioritization by Reduction of Variance’ then you have the option of selecting the type of regression model to use. You have the option of linear, interaction, quadratic, and pure quadratic.
7) Also, if you have selected to perform either ‘Unstandardized Regression’, ‘Standardized Regression’, ‘Logistic regression’, ‘Kolmogorov-Smirnov test’, or ‘Factor Prioritization by Reduction of Variance’, a ‘Load’ button and listbox will also appear. Once you have selected the appropriate parameter and outcome files, you may press the ‘Load’ button. This will gather information from the files, and display a list of the outcome variables in the listbox. You then have a choice as to which outcomes you wish to preform the analysis on, you also have the option to perform it on all outcomes. If you wish to select more than one outcome variable, hold the down the Ctrl button the keyboard and use the mouse to select the variables.

8) Once you have made your selections, press the ‘Run’ button. This will perform the selected sensitivity analysis.

Displayed Data Information:

If you chose to display the output to screen for any of the options above, a pop-up window will appear containing the all of relevant information.

For Pearson, Spearman, and Partial Rank correlation coefficients there are two different types of windows depending on whether or not Office Web Components is installed on your system. Figure 6, shows the non-OWC display. We have four columns of information. The first column is displays the outcome variable and then a list of all the input parameters. If there are multiple outcome variables, the list will repeat itself with the name of the new outcome variable beginning the new list. The second column displays the correlation coefficient associated with the input parameter from the first column. The third column shows the related p-value of each parameter. The last column lists the parameters in order of importance, going from highest to lowest.

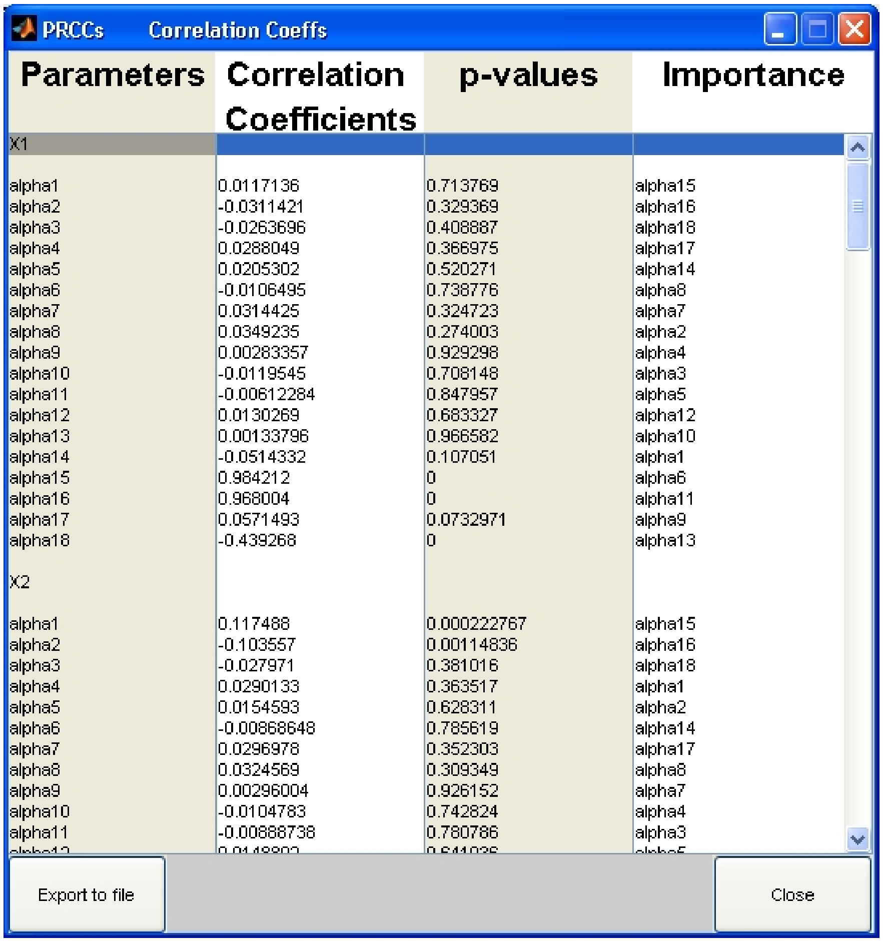

Parameters	Correlation Coefficients	p-values	Importance
X1			
alpha1	0.0117136	0.713769	alpha15
alpha2	-0.0311421	0.329369	alpha16
alpha3	-0.0263696	0.408887	alpha18
alpha4	0.0288049	0.366975	alpha17
alpha5	0.0205302	0.520271	alpha14
alpha6	-0.0106495	0.738776	alpha8
alpha7	0.0314425	0.324723	alpha7
alpha8	0.0349235	0.274003	alpha2
alpha9	0.00283357	0.929298	alpha4
alpha10	-0.0119545	0.708148	alpha3
alpha11	-0.00612284	0.847957	alpha5
alpha12	0.0130269	0.683327	alpha12
alpha13	0.00133796	0.966582	alpha10
alpha14	-0.0514332	0.107051	alpha1
alpha15	0.984212	0	alpha6
alpha16	0.968004	0	alpha11
alpha17	0.0571493	0.0732971	alpha9
alpha18	-0.439268	0	alpha13
X2			
alpha1	0.117488	0.000222767	alpha15
alpha2	-0.103557	0.00114836	alpha16
alpha3	-0.027971	0.381016	alpha18
alpha4	0.0290133	0.363517	alpha1
alpha5	0.0154593	0.628311	alpha2
alpha6	-0.00868648	0.785619	alpha14
alpha7	0.0296978	0.352303	alpha17
alpha8	0.0324569	0.309349	alpha8
alpha9	0.00296004	0.926152	alpha7
alpha10	-0.0104783	0.742824	alpha4
alpha11	-0.00888738	0.780786	alpha3
[illegible]	[illegible]	[illegible]	[illegible]

Figure 6: Example of the non-OWC display.

If you do have OWC installed, then you should see something similar to Figure 7. This has a miniature version of an Excel worksheet embedded in the window. There are three sections, the first sections shows the correlation coefficients, the second shows the p-values, and the third shows the order of importance. The spreadsheet environment allows you to manipulate the data and make any adjustments. Using the button highlighted in Figure 7 by the red circle will automatically open Excel and copy across all the data within the window. Alternatively, you can use the 'Export File' button located on the bottom left of the window to export the data to either an excel file, or to a *.mat file. Note, using this button will not save any changes you have made in the window.

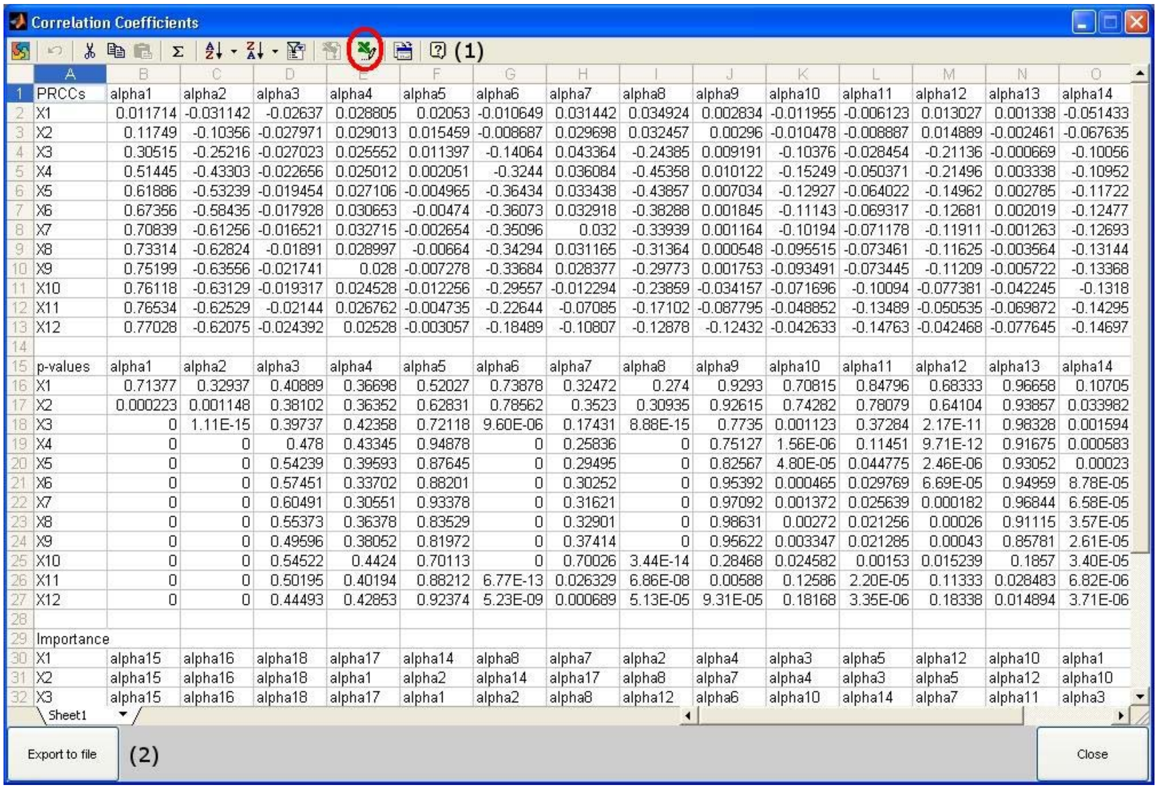

	A	B	C	D	E	F	G	H	I	J	K	L	M	N	O
1	PRCCs	alpha1	alpha2	alpha3	alpha4	alpha5	alpha6	alpha7	alpha8	alpha9	alpha10	alpha11	alpha12	alpha13	alpha14
2	X1	0.011714	-0.031142	-0.02637	0.028805	0.02053	-0.010649	0.031442	0.034924	0.002834	-0.011955	-0.006123	0.013027	0.001338	-0.051433
3	X2	0.11749	-0.10356	-0.027971	0.029013	0.015459	-0.008687	0.029698	0.032457	0.00296	-0.010478	-0.008887	0.014889	-0.002461	-0.067635
4	X3	0.30515	-0.25216	-0.027023	0.025552	0.011397	-0.14064	0.043364	-0.24385	0.009191	-0.10376	-0.028454	-0.21136	-0.000669	-0.10056
5	X4	0.51445	-0.43303	-0.022656	0.025012	0.002051	-0.3244	0.036084	-0.45358	0.010122	-0.15249	-0.050371	-0.21496	0.003338	-0.10952
6	X5	0.61886	-0.53239	-0.019454	0.027106	-0.004965	-0.36434	0.033438	-0.43857	0.007034	-0.12927	-0.064022	-0.14962	0.002785	-0.11722
7	X6	0.67356	-0.58435	-0.017928	0.030653	-0.00474	-0.36073	0.032918	-0.38288	0.001845	-0.11143	-0.069317	-0.12681	0.002019	-0.12477
8	X7	0.70839	-0.61256	-0.016521	0.032715	-0.002654	-0.35096	0.032	-0.33939	0.001164	-0.10194	-0.071178	-0.11911	-0.001263	-0.12693
9	X8	0.73314	-0.62824	-0.01891	0.028997	-0.00664	-0.34294	0.031165	-0.31364	0.000548	-0.095515	-0.073461	-0.11625	-0.003564	-0.13144
10	X9	0.75199	-0.63556	-0.021741	0.028	-0.007278	-0.33684	0.028377	-0.29773	0.001753	-0.093491	-0.073445	-0.11209	-0.005722	-0.13368
11	X10	0.76118	-0.63129	-0.019317	0.024528	-0.012256	-0.29557	-0.012294	-0.23859	-0.034157	-0.071696	-0.10094	-0.077381	-0.042245	-0.1318
12	X11	0.76534	-0.62529	-0.02144	0.026762	-0.004735	-0.22644	-0.07085	-0.17102	-0.087795	-0.048852	-0.13489	-0.050535	-0.069872	-0.14295
13	X12	0.77028	-0.62075	-0.024392	0.02528	-0.003057	-0.18489	-0.10807	-0.12878	-0.12432	-0.042633	-0.14763	-0.042468	-0.077645	-0.14697
14															
15	p-values	alpha1	alpha2	alpha3	alpha4	alpha5	alpha6	alpha7	alpha8	alpha9	alpha10	alpha11	alpha12	alpha13	alpha14
16	X1	0.71377	0.32937	0.40889	0.36698	0.52027	0.73878	0.32472	0.274	0.9293	0.70815	0.84796	0.68333	0.96658	0.10705
17	X2	0.000223	0.001148	0.38102	0.36352	0.62831	0.78562	0.3523	0.30935	0.92615	0.74282	0.78079	0.64104	0.93857	0.033982
18	X3	0	1.11E-15	0.39737	0.42358	0.72118	9.60E-06	0.17431	8.88E-15	0.7735	0.001123	0.37284	2.17E-11	0.98328	0.001594
19	X4	0	0	0.478	0.43345	0.94878	0	0.25836	0	0.75127	1.56E-06	0.11451	9.71E-12	0.91675	0.000583
20	X5	0	0	0.54239	0.39593	0.87645	0	0.29495	0	0.82567	4.80E-05	0.044775	2.46E-06	0.93052	0.00023
21	X6	0	0	0.57451	0.33702	0.88201	0	0.30252	0	0.95392	0.000465	0.029769	6.69E-05	0.94959	8.78E-05
22	X7	0	0	0.60491	0.30551	0.93378	0	0.31621	0	0.97092	0.001372	0.025639	0.000182	0.96844	6.58E-05
23	X8	0	0	0.55373	0.36378	0.83529	0	0.32901	0	0.98631	0.00272	0.021256	0.00026	0.91115	3.57E-05
24	X9	0	0	0.49596	0.38052	0.81972	0	0.37414	0	0.95622	0.003347	0.021285	0.00043	0.85781	2.61E-05
25	X10	0	0	0.54522	0.4424	0.70113	0	0.70026	3.44E-14	0.28468	0.024582	0.00153	0.015239	0.1857	3.40E-05
26	X11	0	0	0.50195	0.40194	0.88212	6.77E-13	0.026329	6.86E-08	0.00588	0.12586	2.20E-05	0.11333	0.028483	6.82E-06
27	X12	0	0	0.44493	0.42853	0.92374	5.23E-09	0.000689	5.13E-05	9.31E-05	0.18168	3.35E-06	0.18338	0.014894	3.71E-06
28															
29	Importance														
30	X1	alpha15	alpha16	alpha18	alpha17	alpha14	alpha8	alpha7	alpha2	alpha4	alpha3	alpha5	alpha12	alpha10	alpha1
31	X2	alpha15	alpha16	alpha18	alpha1	alpha2	alpha14	alpha17	alpha8	alpha7	alpha4	alpha3	alpha5	alpha12	alpha10
32	X3	alpha15	alpha16	alpha18	alpha17	alpha1	alpha2	alpha8	alpha12	alpha6	alpha10	alpha14	alpha7	alpha11	alpha3

Figure 7: Example of displayed output for correlation coefficients if OWC is installed. (1) toolbar featuring various Excel type commands, (2) 'Export' button to save information to a file. The red circle indicates the 'Export to Excel' function, pressing this button will open Excel and transfer all the data currently contained with in the worksheet.

For unstandardized and standardized regression, the display consists of two sections. The first section is displayed in the top right corner and shows the coefficients of determination for each output variable. The second section takes up most of the window, and is made up of four columns. The first column gives a list the outcome variable, followed by each parameter. For example, in Figure 8, the first outcome variable is 'X1', underneath that all the parameters are listed. The next outcome variable is 'X2' and is seen after the first list of parameters.

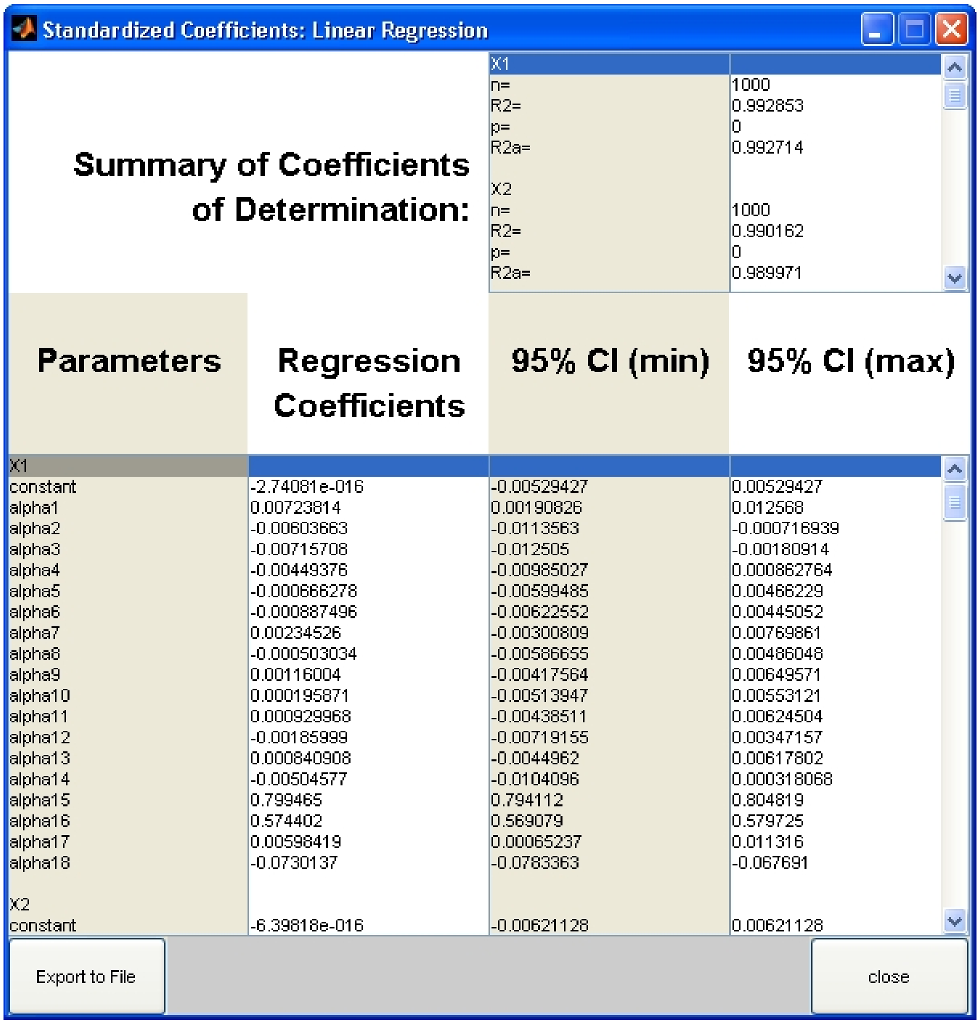

Parameters	Regression Coefficients	95% CI (min)	95% CI (max)
X1			
constant	-2.74081e-016	-0.00529427	0.00529427
alpha1	0.00723814	0.00190826	0.012568
alpha2	-0.00603663	-0.0113563	-0.000716939
alpha3	-0.00715708	-0.012505	-0.00180914
alpha4	-0.00449376	-0.00985027	0.000862764
alpha5	-0.000666278	-0.00599485	0.00466229
alpha6	-0.000887496	-0.00622552	0.00445052
alpha7	0.00234526	-0.00300809	0.00769861
alpha8	-0.000503034	-0.00586655	0.00486048
alpha9	0.00116004	-0.00417564	0.00649571
alpha10	0.000195871	-0.00513947	0.00553121
alpha11	0.000929968	-0.00438511	0.00624504
alpha12	-0.00185999	-0.00719155	0.00347157
alpha13	0.000840908	-0.0044962	0.00617802
alpha14	-0.00504577	-0.0104096	0.000318068
alpha15	0.799465	0.794112	0.804819
alpha16	0.574402	0.569079	0.579725
alpha17	0.00598419	0.00065237	0.011316
alpha18	-0.0730137	-0.0783363	-0.067691
X2			
constant	-6.39818e-016	-0.00621128	0.00621128

Figure 8: Example of displayed output from Standardized Regression. In this example the first outcome variable is 'a1' and the second is 'a4'.

Logistic regression is outputted in a similar way to the previous regression analyses. The summary of the coefficients of determination is again display in the top right-hand corner. The display shows the outcome variable name, the R^2 value, and the number of results. Below this, there are six columns of information. The first column lists the outcome variable name, followed by a list of each parameter. If there are multiple outcome variables, the list will cycle again, but with the new outcome name denoting the start of the next list. The next several columns represent data associated with each parameter, these are: Beta Coefficient, p-value, Odds Ratio, and the 95% confidence interval.

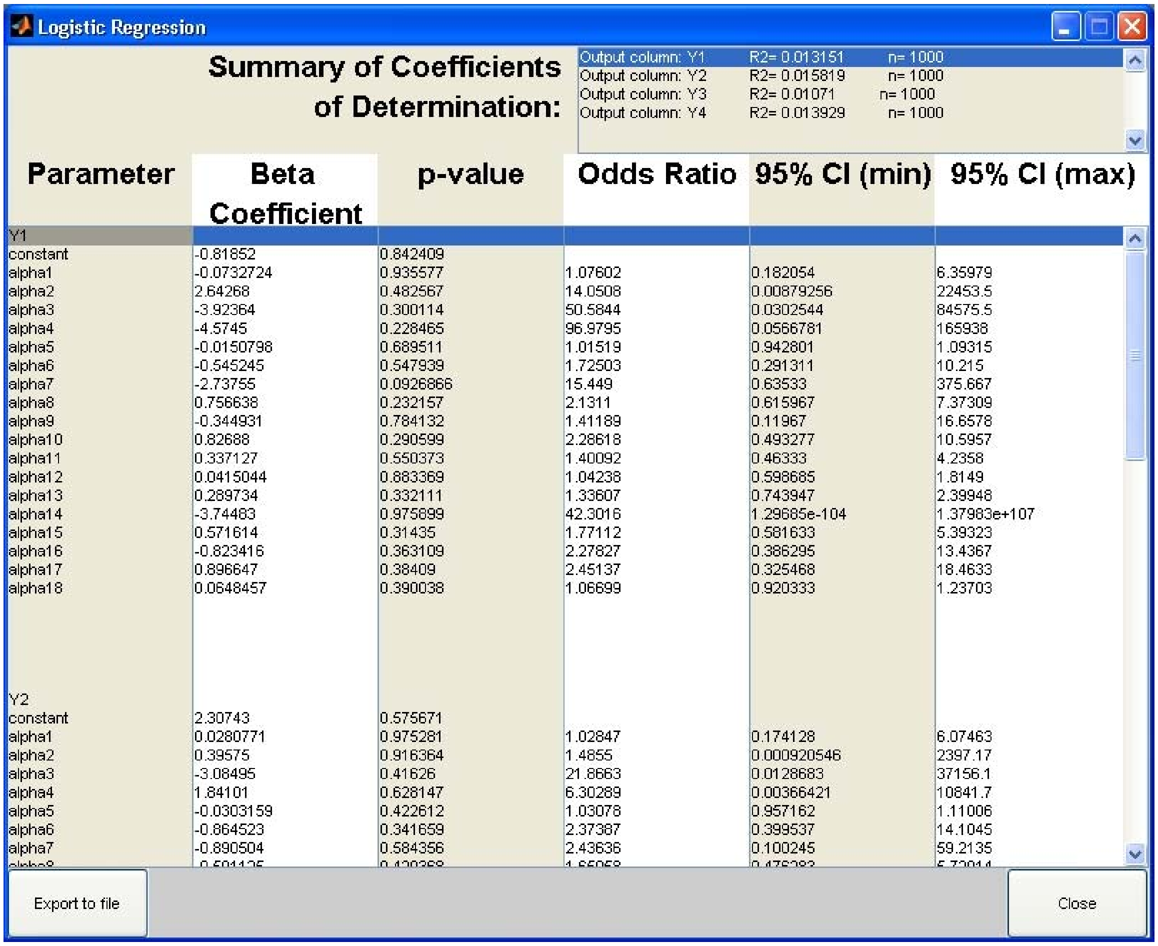

Parameter	Beta Coefficient	p-value	Odds Ratio	95% CI (min)	95% CI (max)
Y1					
constant	-0.81852	0.842409			
alpha1	-0.0732724	0.935577	1.07602	0.182054	6.35979
alpha2	2.64268	0.482567	14.0508	0.00879256	22453.5
alpha3	-3.92364	0.300114	50.5844	0.0302544	84575.5
alpha4	-4.5745	0.228465	96.9795	0.0566781	165938
alpha5	-0.0150798	0.689511	1.01519	0.942801	1.09315
alpha6	-0.545245	0.547939	1.72503	0.291311	10.215
alpha7	-2.73755	0.0926866	15.449	0.63533	375.667
alpha8	0.756638	0.232157	2.1311	0.615967	7.37309
alpha9	-0.344931	0.784132	1.41189	0.11967	16.6578
alpha10	0.82688	0.290599	2.28618	0.493277	10.5957
alpha11	0.337127	0.550373	1.40092	0.46333	4.2358
alpha12	0.0415044	0.883369	1.04238	0.598685	1.8149
alpha13	0.289734	0.332111	1.33607	0.743947	2.39948
alpha14	-3.74483	0.975899	42.3016	1.29685e-104	1.37983e+107
alpha15	0.571614	0.31435	1.77112	0.581633	5.39323
alpha16	-0.823416	0.363109	2.27827	0.386295	13.4367
alpha17	0.896647	0.38409	2.45137	0.325468	18.4633
alpha18	0.0648457	0.390038	1.06699	0.920333	1.23703
Y2					
constant	2.30743	0.575671			
alpha1	0.0280771	0.975281	1.02847	0.174128	6.07463
alpha2	0.39575	0.916364	1.4855	0.000920546	2397.17
alpha3	-3.08495	0.41626	21.8663	0.0128683	37156.1
alpha4	1.84101	0.628147	6.30289	0.00366421	10841.7
alpha5	-0.0303159	0.422612	1.03078	0.957162	1.11006
alpha6	-0.864523	0.341659	2.37387	0.399537	14.1045
alpha7	-0.890504	0.584356	2.43636	0.100245	59.2135
[illegible]	[illegible]	[illegible]	[illegible]	[illegible]	[illegible]

Figure 9: Example of data displayed from a Logistic Regression analysis. The upper left corner provides a summary of the Coefficients of Determination for each of the outcome variables.

The Kolmogorov-Smirnov test produces a similar display. As seen in Figure 10, it is comprised of five columns. The first column, as with the others, is used to display the parameter names and outcome variables. The second column is the importance rank. This orders each parameter in order of its influence on the outcome variable. The third column highlights the most influential parameters, with ones representing parameters with a significant influence. The fourth column displays the p-value associated with each parameter and outcome variable. The final column reports the greatest difference between the cumulative distribution functions.

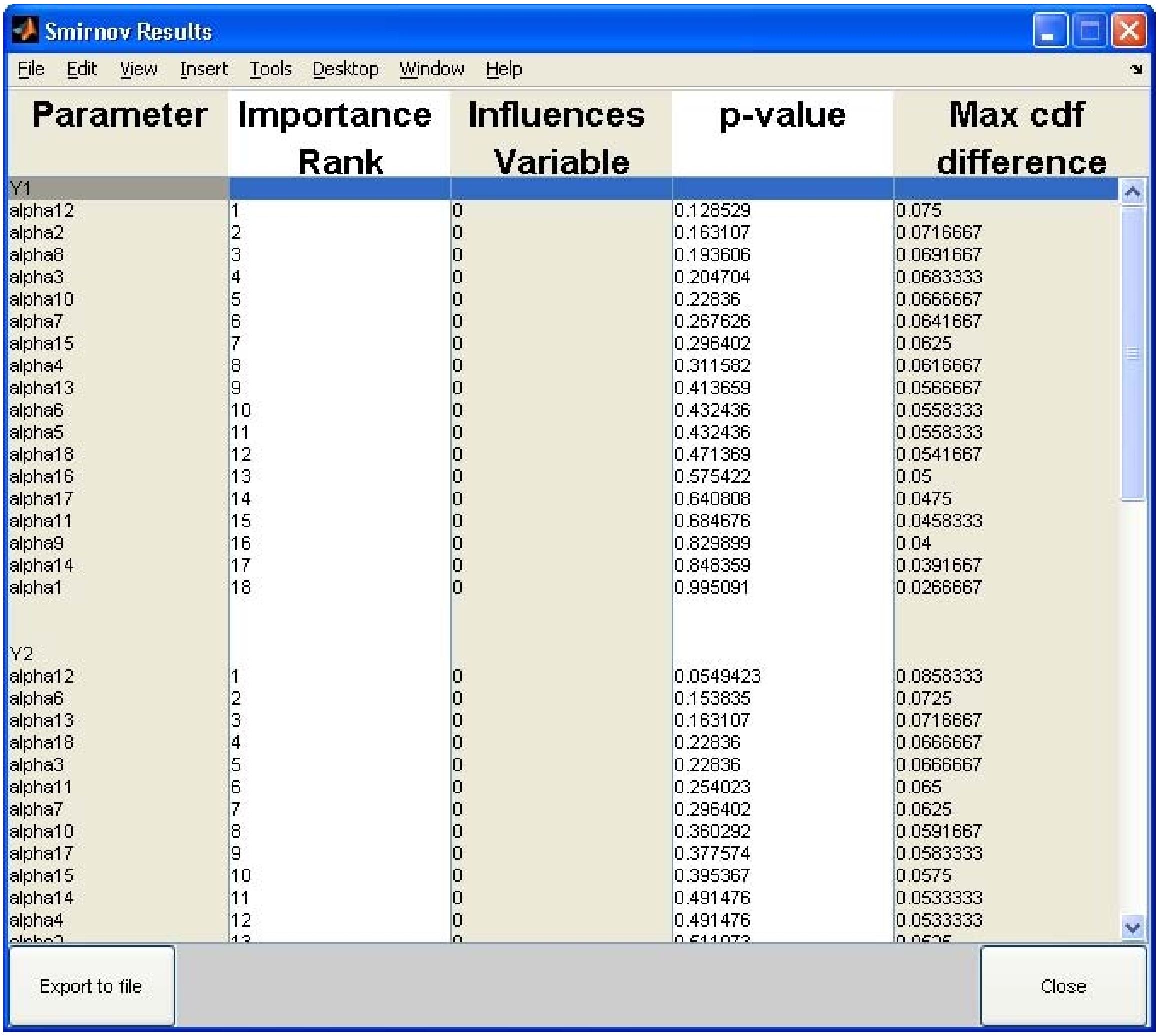
Smirnov Results

File Edit View Insert Tools Desktop Window Help

Parameter	Importance Rank	Influences Variable	p-value	Max cdf difference
Y1				
alpha12	1	0	0.128529	0.075
alpha2	2	0	0.163107	0.0716667
alpha8	3	0	0.193606	0.0691667
alpha3	4	0	0.204704	0.0683333
alpha10	5	0	0.22836	0.0666667
alpha7	6	0	0.267626	0.0641667
alpha15	7	0	0.296402	0.0625
alpha4	8	0	0.311582	0.0616667
alpha13	9	0	0.413659	0.0566667
alpha6	10	0	0.432436	0.0558333
alpha5	11	0	0.432436	0.0558333
alpha18	12	0	0.471369	0.0541667
alpha16	13	0	0.575422	0.05
alpha17	14	0	0.640808	0.0475
alpha11	15	0	0.684676	0.0458333
alpha9	16	0	0.829899	0.04
alpha14	17	0	0.848359	0.0391667
alpha1	18	0	0.995091	0.0266667
Y2				
alpha12	1	0	0.0549423	0.0858333
alpha6	2	0	0.153835	0.0725
alpha13	3	0	0.163107	0.0716667
alpha18	4	0	0.22836	0.0666667
alpha3	5	0	0.22836	0.0666667
alpha11	6	0	0.254023	0.065
alpha7	7	0	0.296402	0.0625
alpha10	8	0	0.360292	0.0591667
alpha17	9	0	0.377574	0.0583333
alpha15	10	0	0.395367	0.0575
alpha14	11	0	0.491476	0.0533333
alpha4	12	0	0.491476	0.0533333
[illegible]	[illegible]	[illegible]	[illegible]	[illegible]

Export to file | Close

Figure 10: Example of displayed output from the Kolmogorov-Smirnov test.

Results from the ‘Factor Prioritization by Reduction of Variance’ analyses have only two columns, the parameters and the sensitivity index. The first column is the same as with previous displays, with the outcome variable being listed first, followed by each parameter. The sensitivity index represents the amount of variability in a particular outcome variable that is attributable to each input parameter. For example, looking at Figure 11, the parameter ‘alpha15’ accounts for around 65.6% of the variability in the outcome variable ‘X1’.

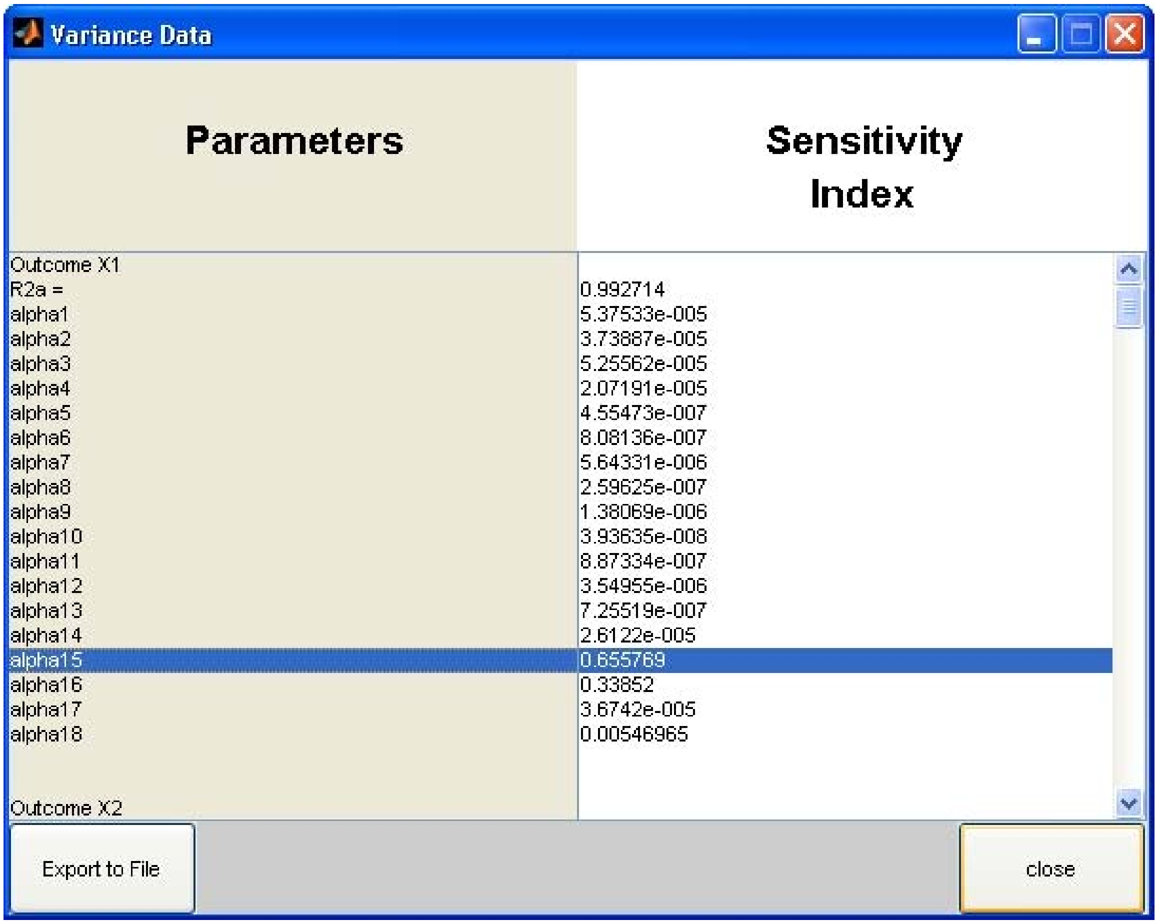

Figure 11: Example of displayed output from the Reduction of Variance analysis.

Chapter 5: Sensitivity Analysis Plots

Plots are an excellent way to visually display data. As such, SaSAT provides several plotting functions to help display the data from a sensitivity analysis.

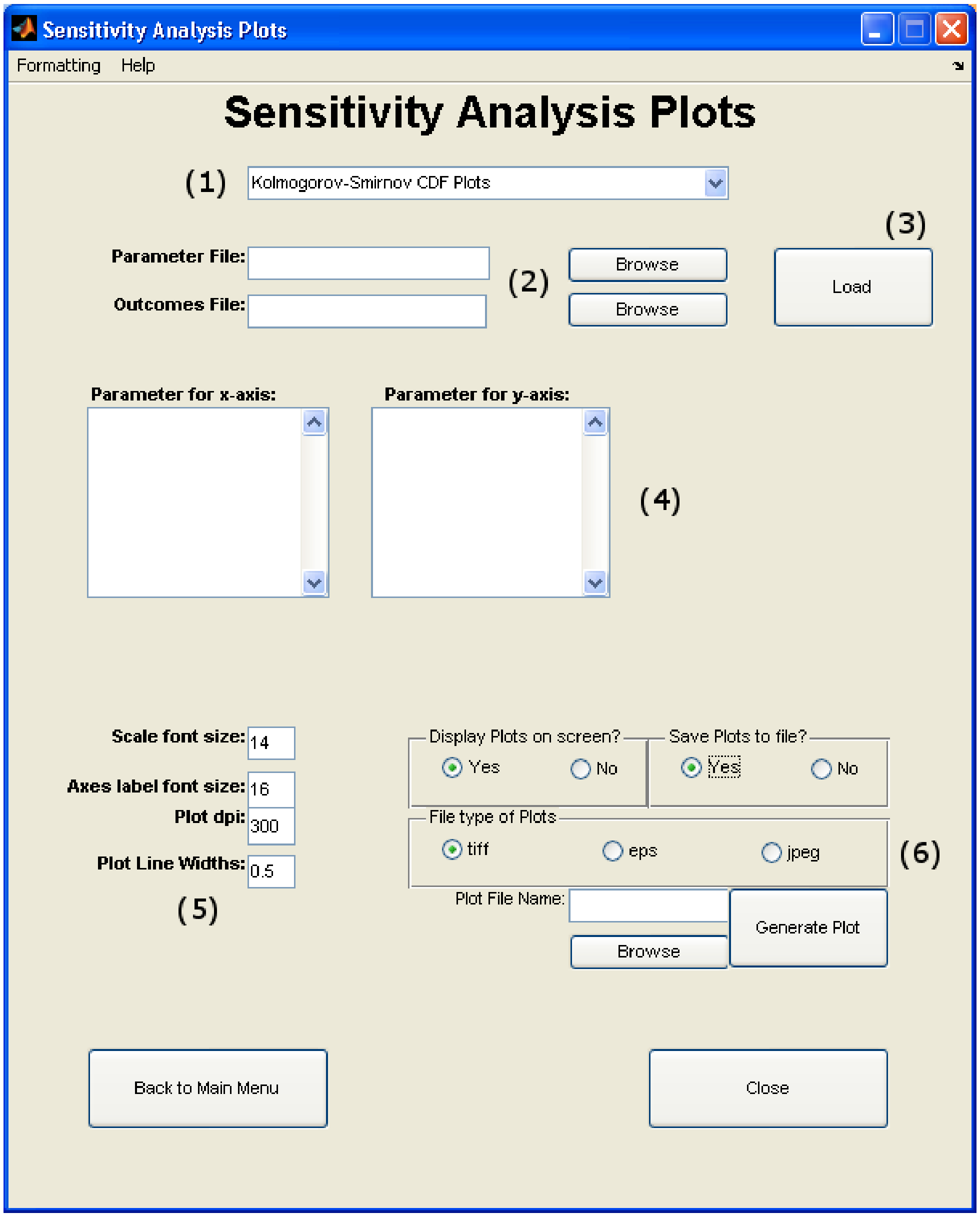

Figure 12: (1) Drop down menu for selecting a plot type (step 1), (2) text fields and browse buttons for file selection (step 2), (3) Load button (step 3), (4) Listboxes that display parameter or outcome names (step 4). (5) Plot options (step 5), (6) Plot output options and Generate button (steps 6-7).

Step-by-Step Guide (refer to Figure12):

1) Select the type of plot from the drop down menu
2) Depending on the type of plot you have selected, there will be different file selection options.
    a. Scatter plots – you will need to select a Parameter file and an Outcomes file. This can be done by typing the name of the file in the text field or by selecting the files using the 'Browse' buttons.
    b. Tornado plots – for this type of plot you will need to select an Analysis file. This type of file is generated from Pearson, Spearman or Partial Rank correlation coefficient sensitivity analyses from the previous chapter.
    c. Response Surface – same as Scatter plots.
    d. Box Plots – you will need to select a file with the structure as described in Chapter 6. Also, here you may enter titles for the x and y axis.
    e. Pie Chart – requires the analysis file created from the Factor Prioritization by Reduction of Variance sensitivity analysis.
    f. Cumulative Distribution plots – similar to the Tornado plot options, but the file structure is the same as for Box plots.
    g. Kolmogorov-Smirnov plots – Same as for Scatter plots.
3) Once you have selected the files required for you plot, you may have to then load the parameter or outcome names into the listboxes (for all plot types except box plots).
4) Once the names of parameters and outcome variables have loaded, you will need to make some selections. This depends on which type of plot you have chosen:
    a. Scatter plots – you will need to choose a parameter for the x-axis, and an outcome variable for the y-axis.
    b. Tornado plots – you only need to select one outcome variable.
    c. Response Surface – you must select a parameter for the x and y axes, and an outcome variable for the response axis. You will also need to select which type of regression you wish to use via the radio buttons underneath the listboxes.
    d. Box plots – requires no selection.
    e. Pie Chart – this only requires you to choose an outcome variable.
    f. Cumulative Distribution plots – select which outcomes that you wish to see. To select multiple outcomes, hold 'Ctrl' and click on the outcomes.
    g. Kolmogorov-Smirnov plots – you must choose an outcome, and the input parameter. You are also able to select multiple parameters by holding the Ctrl key and clicking the parameter names. This will produce multiple plots.
5) When you have picked the appropriate variable titles, you will now need to set some of the figure options. There are different options for different types of plots, but they all share the options to set the font size for items on the axes scale, setting the font size for axes labels, and the plot dpi. Note, that for a Pie Chart, you are unable to change to font sizes but are still able to set the resolution. Plot specific options are:

a. Scatter plots:
    i. Scatter plot symbol – this allows you to select the symbol to be displayed on screen via a drop down menu.
    ii. Symbol size – allows you to set the size of the symbols.
b. Tornado plots:
    i. CC cut off – this option lets you set a cut off level on the correlation coefficient. That is, you can choose to plot only the parameters with correlation coefficients above the specified level.
c. Response Surface:
    i. Number of Colors – increases the number of colors used for the surface plot. Has the same effect of increasing the resolution of the response axes.
    ii. Min response shown – allows you to set the minimum value of the surface. Default is set to 'auto' this will use the minimum value calculated by the program.
    iii. Max response shown – allows you to set the maximum value of the surface. Default is set to 'auto' this will use the maximum value calculated by the program.
d. Box plots:
    i. Whisker length – sets the length of the box whiskers. This number is in units of the IQR. For example, the default of 1.5 means that the whisker length will be 1.5 multiplied by the inter-quartile range, and any values outside this maximum whisker length will be displayed as outlier points.
e. Pie Chart:
    i. Percentage cut off – this option is similar to the CC cut off for Tornado Plots in that any parameters that contribute less than the set value to the variability, will be lumped together in an 'other' category. The default value is 0.05, which means that any parameters that contribute less than 5% to the variability will be grouped together.
f. Cumulative Distribution plots:
    i. Plot line widths – changes the line width of the CDF lines.
g. Kolmogorov-Smirnov plots:
    i. Plot line widths – change the line widths for the plot.

6) Next, you must choose whether or not to display or print the plots, or do both. This is done by selecting the appropriate options via the radio buttons. If you choose to save the plots to a file, you will be given the option of choosing the file type and picking the file name.
7) When you are ready, press the 'Generate Plot' button to run. If the display plot option is set, the plot figures will pop up. If you chose to save the files you will receive a message telling you that the file was saved successfully.

Chapter 6: Other Tools

File Converter

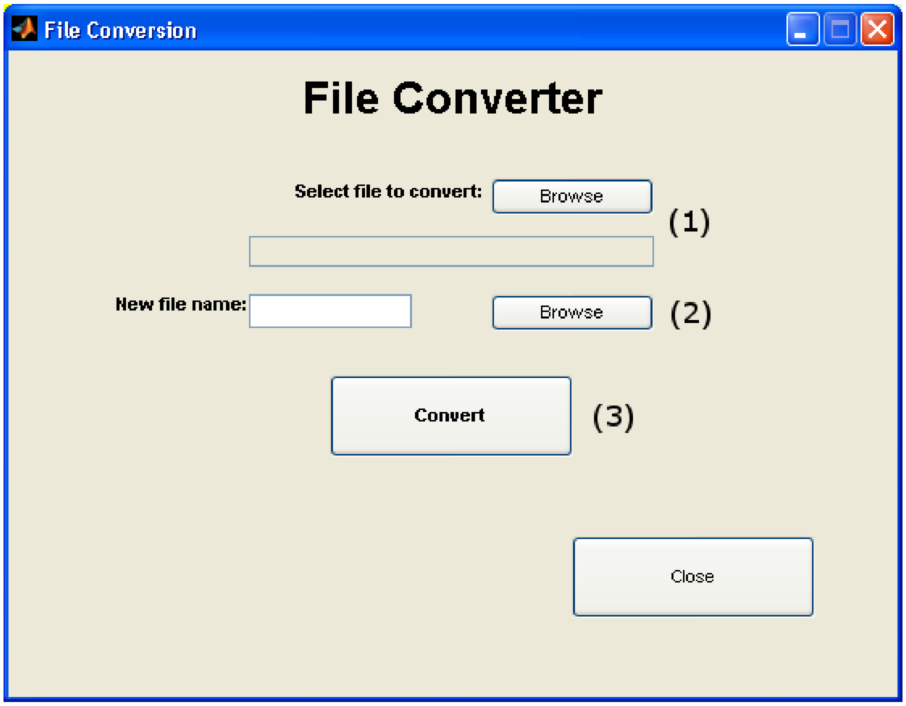

Figure 13: (1) Browse button and text field (step 1), (2) area to select the destination of the converted file (3) Convert button will begin the file conversion process (step 3).

Because SaSAT relies heavily on two file formats (*.xls, and *.mat), we have included a file converting tool to allow you to move easily from one format to the other. This can be accessed via the menu bar on any of the utility pages, under the 'Formatting' tab.

To use:

1) Select file to be converted, you can select either a *.mat file or a *.xls file. Below the 'Browse' button, is a text field that cannot be edited and is only used to display your file selection.
2) Once a file for conversion has been selected, a destination file must be selected. The type of file to be created is depends on the type of file that is selected in step 1. For example, if an *.xls file was selected, a *.mat file will be created.
3) After both files have been selected, pressing the 'Convert' button will begin the conversion process. A message box will then appear to let you know that the conversion process has been complete.

Mat File Formatter

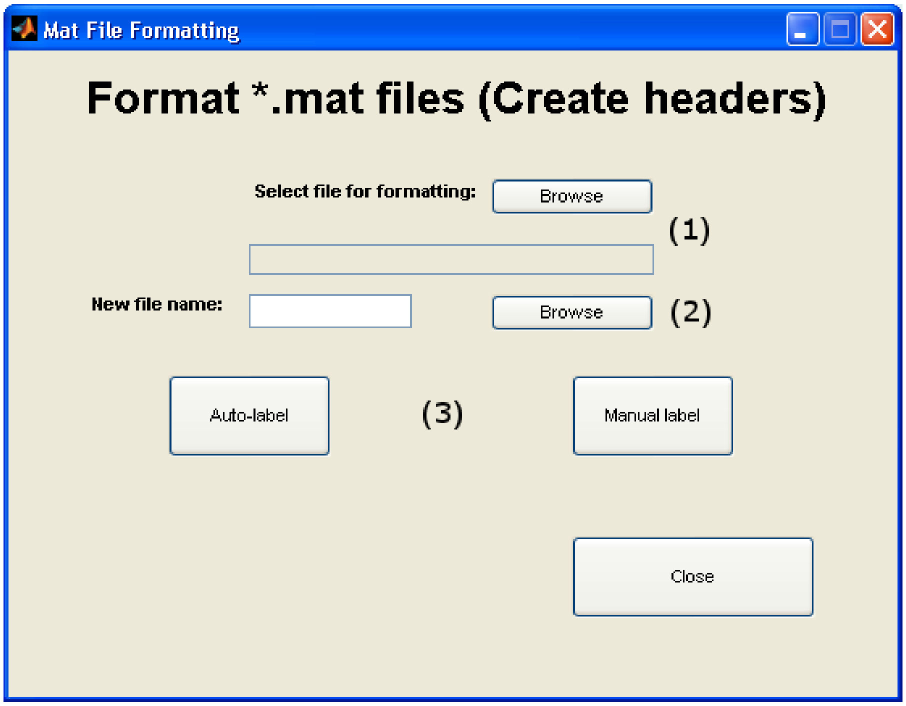

Figure 14: (1) Browse button and text field (step 1), (2) area to select the destination of the formatted file, (3) buttons to select either Auto-label, or Manual label (step 3).

Another useful tool that we have included with SaSAT is the ability to add column labels in *.mat files. Since SaSAT requires output data to be in a particular format (see Chapter 7), with titles for each column and manipulating *.mat files is more difficult than it is with Excel, we have created an easy to use GUI. This tool can be accessed in a similar way to the File Conversion tool, on the menu bar under the 'Formatting' tab.

To use:

1) Select *.mat file to be formatted. The directory path will be displayed in the text field below.
2) Select the destination file name and location using the 'Browse' button.
3) Once both files have been selected, you have the option to Auto-label the columns, or use Manual label. Auto-label will automatically assign each column the title 'Output 1, Output 2, Output 3… Output x', where there are x number of columns. Manual label enables you to enter the headings for each column.

Chapter 7: Notes about file structure

The structure of the various input files are very important, as the program files rely on some data being placed in specific places within the file. If the files are not of the right structure, you may receive errors. Files that are created by our program will automatically have the correct structure. Only the output data file generated by your models may not fit the required template, which is why we shall provide some examples of how these files are set up.

Definition File:

Parameter Name	Distribution	Output (1/0)	Argument 1	Argument 2	Argument 3
param1	1	1	0	1	
…	…	…	…	…	…

This is how the definition file is structured. All parameter names are in the first column, the second column contains a number that specifies the distribution type used. The third column displays either a 1 or a 0 to show if this parameter is to be outputted or not. The final three columns are for the arguments of the distribution type (see Table 1). If you create a definitions file via the Parameter Definition page (see Chapter 2), the created file will automatically have this structure. If you choose to create the file yourself, make sure that the first line contains the titles shown above. In this example, param1 has a uniform distribution, will be outputted, and had minimum and maximum values of 0 and 1 respectively.

Parameter File:

param1	param2	param3	…
…	…	…	…

In this file, the parameter names are displayed across the first row. Each row after that corresponds to the parameter values for one simulation of a model. A file with this structure will be created if you use the Generate Samples page (see Chapter 3).

Outcomes File:

Outcome 1	Outcome 2	Outcome 3	…
…	…	…	…

When building your model, take into account how the data it generates is outputted. For use with our software, it is best the output of each cycle is placed in a row, and each column represents an outcome variable. It is important to have names of each outcome variable displayed in the first row, as SaSAT will need to read in these names to function properly. In Excel this can be done by simply shifting the data down by one row, and

typing the outcome names in the cell in the appropriate column. If you are using *.mat files, then manipulating the data can be trickier. However, SaSAT includes a simple tool to help add column titles for output files (see Chapter 6).

Tornado Plot File:

Correlation Type	param1	param2	…
Outcome1	…	…	…
Outcome2	…	…	…
…	…	…	…

The tornado plot file should be of the same structure as the output file from a correlation coefficients sensitivity analyses. That is, all input parameters are listed in the first row, starting from the second column. Also, all outcome variable names are listed in the first column starting from the second row. The rest of the cells should contain the correlation coefficients.

Box Plot File:

Outcome 1a	Outcome 1b	Outcome 1c	…
…	…	…	…

This is similar to the layout of outcomes file, and in fact the outcomes file will work with the box plot program. However, it is better to use the box plots to compare similar data. For example, it would typically be used to compare the results of several parameter sets.

Pie Chart File:

Outcome	Outcome 1	Outcome 2	Outcome 3	…
R2a	…	…	…	…
Parameter 1	…	…	…	…
Parameter 2	…	…	…	…
…	…	…	…	…

This is the file structure that is produced when you save the data from a Factor Prioritization by Reduction of Variance sensitivity analysis. The outcome variables are listed along the 1st row, starting from the second column. The input parameters are listed in the first column, starting from the 3rd row. The adjusted R squared value fills the 2nd row, but is ignored in the Pie Chart code.

CDF Plot File:

This type of plot is very similar to the boxplot, and as a result can use the same file. Again, it is best used for comparing similar outcome variables, or the same outcome variable generated from different parameter sets.

Chapter 8: List of Error messages

Parameter Definitions:

`Error: You must specify a file extension, either *.mat, or *.xls` – occurs when you enter a filename into the text field (see Figure 2, item (9)), and do not specify a file type. To rectify, a file extension must be added, i.e. MyFilename → MyFilename.mat.

`Error: Unrecognized distribution type.  Please check file.` – occurs if there is an error in the file selected in step 1 of Chapter 2, (see Figure 2, item (1)). The program was unable to find a recognized distribution type. To rectify, manually check the file for errors, consult Table 1 for list of known distribution types.

`Error: Input file is not of correct format.  Please check file.` – occurs if the selected distribution file is not of the correct format. To rectify, check that the selected file is of the same format as given in Chapter 6.

`Error: Unrecognized output type.  Please check file.` – similar to the unrecognized distribution type error above.

Sample Generation:

`Error: Please select a sampling technique.` – a sampling technique was not selected from the drop down menu (see Figure 3, item (1)).

`Error: You must enter a number of samples.` – occurs if no number is entered in the text field for selecting the number of samples (Figure 3, item (7)). Also occurs if this number is 0.

`Error: Output file was not saved correctly.` – this error occurs if the selected output file was not created successfully. This can be due to a number of things. First check that you have selected appropriate filenames. Another thing to check is, if you are working with an Excel file, make sure that you do not have that file with the chosen output name open. Just close this file and run the program again.

`Error: Definition file is not of the right format. Please check this file is correct.` – this error will appear if the selected definition file is not in the correct format. Check that you have selected the correct definition file. If this still occurs, check that the definition file is set out according to Chapter 7.

`Error: Incorrect number of parameters for distribution.` – this message will appear if the definition file contains an incorrect number of arguments. To fix, check

the definition file and make sure that each distribution has the correct number of arguments shown in Table 1.

`Error: A distribution has been encountered for a dependent variable that is incorrectly defined.` – this error will occur due to an incorrect number in the definition file. The incorrect number will be in the Distribution column, for dependant variables the distribution numbers must be between 21 and 27.

`Error: No parameters given for this distribution.` – occurs if the parameters have been incorrectly defined for a given distribution. Check the definition file for any errors.

`Error: Unknown distribution.` – the distribution type was not recognized. Check the definition file to ensure that the distribution numbers are within the range of 0 – 8 for independent parameters, and 21 – 27 for dependent parameters.

Sensitivity Analysis

`Error: The Parameter or Outcome file that you have specified does not exist. Please check your selection.` – the file names that were inputted were not correct. Try using the 'Browse' button (Figure 5, item (2)) to select a file.

`Error: One or more of the required filename fields is empty. Please check that a filename has been entered.` – this error occurs if one of the necessary text fields is empty (Figure 5, item (2)). Make sure that you have selected a correct file and run the program again.

`Error: The Outcomes File text field is empty. Please select/enter a file name.` – as with the above, make sure that you have selected or entered a correct filename.

`Error: No such file exists in the specified location. Please select another file.` – if this occurs, then check that the file you have selected exists. If you have manually entered the filename into the text field, make sure that the file is located in the same directory as the program. Alternatively you can use the 'Browse' button to select the correct file.

`Error: Outcomes file is not of the correct format. Please check this file.` – this error will occur if your output file is not structured in the correct way. Please see Chapter 7 for examples of correct file setups.

`Error: Output file was not created successfully.` – similar to the other file creation errors.

`Error: Parameter file and outcome file do not have compatible dimensions.` – this error will occur if the size of the input parameter file is not the

same as the outcomes file. That is, the number of simulations is not the same. Check both files visually.

`Error: Outcomes file incorrect must contain only 1''s and 0''s.` – this error applies to Logistic Regression and the Kolmogorov-Smirnov test. The outcomes file for these two sensitivity analyses must be made up of vectors of 1's and 0's. Check that you have selected the correct outcome file, and see Chapter 7 for more details on file structure.

Sensitivity Plots

`Error: Filename missing or not valid.` – this will appear if the 'Load' button is pressed without any filename(s) being specified (see Figure 12, item (3)).

`Error: Incorrect input argument. Please check that Font size and resolution fields contain numerical values.` – occurs if no numbers have been set for the axes, and scale font size, or the plot resolution. Make sure to enter appropriate values and run the program again (see Figure 12, item (5)).

`Error: You must load some variables first. Select a file and press the Load button.` – this error will occur if you have chosen to run the program without first loading variables into the listboxes (see Chapter 5, step 3).

`Error: You must select a data file first.` – this error will occur if you have not selected a parameter file for the Box plot option. Make sure that a file has been selected before proceeding with the program.

`Error: You must specify a file name to save your plot.` – if you have chosen to save a plot, then you must enter a filename to save the plot under.

Chapter 9: Glossary

GUI – Graphical User Interface, which is the collection of buttons, listboxes, text fields, etc that allows the user to interact with the program.

Listbox (Figure 15) – a GUI object that displays a list of items on the screen. It allows the user to highlight and select items displayed in the list. Sometimes multiple items can be highlighted by holding the Control button, or by clicking and dragging the mouse.

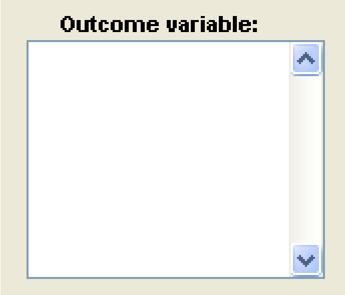

Figure 15: An example of a listbox

Text field (Figure 16) – an object embedded within the GUI that allows users to enter a text string.

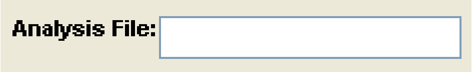

Figure 16: Example of a text field

Drop down menu (Figure 17) – a GUI object that when clicked by the user brings up a list of items. The user can then select an item with the mouse.

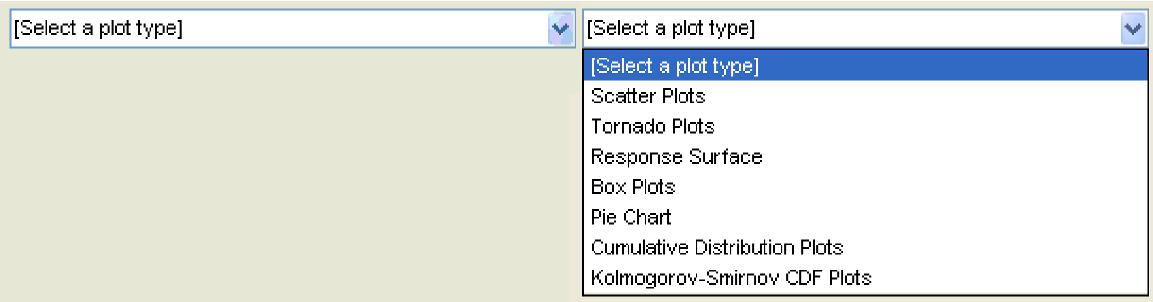

Figure 17: Example of a drop down menu, before and after it has been selected.

Radio Buttons (Figure 18) – a GUI object that is either 'on' or 'off', the user can set this by clicking on the button.

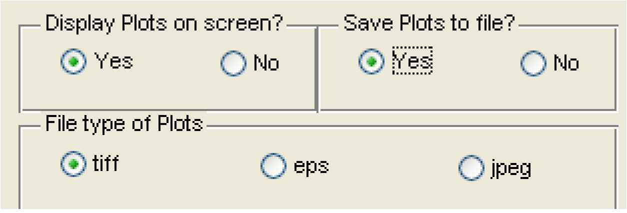

Figure 18: An example of a group of radio buttons

Outcome variable – an outcome variable is used to describe the variable obtained from the output of a model. This will be data that you have created using the input parameters generated in SaSAT, for your models.

Input Parameter – these are the parameters used in your model, which have to be defined before the model is run.
